# Stable Carbon and Oxygen Isotope Analysis of Carbonates and DIC Using the Delta Ray Isotope Ratio Infrared Spectrometer (IRIS): Precise and Accurate Measurements Applying a 3‐Point Calibration and Standard Bracketing

**DOI:** 10.1002/rcm.70021

**Published:** 2025-12-29

**Authors:** Maximilian Hansen, Jakob Brettschneider, Hubert Vonhof, Denis Scholz

**Affiliations:** ^1^ Institute for Geosciences University of Mainz Mainz Germany; ^2^ Max‐Planck‐Institute for Chemistry Mainz Germany

**Keywords:** 3‐point calibration for stable isotopes, optical isotope ratio spectroscopy, OIRS, optical measurement of δ^13^C and δ^18^O on CO_2_, carbonate and DIC, standard bracketing, standard calibration

## Abstract

**Rationale:**

The Delta Ray Isotope Ratio Infrared Spectrometer enables to determine δ^13^C and δ^18^O values of CO_2_. The measurements are calibrated against internal reference gas standards. For discrete carbonate or dissolved inorganic carbon (DIC) samples, the manufacturer recommends a calibration against two isotopically different carbonate standard materials (i.e., a 2‐point calibration). Here, we show that this method is not sufficient because the measured values may drift significantly over the duration of a measurement run (i.e., 58 samples in approximately 24 h).

**Methods:**

We developed a new measurement routine applying a 3‐point calibration and a tight standard‐bracketing. In addition, we present an R code to automatically evaluate and calibrate the measured data.

**Results:**

Our standard‐bracketing technique can effectively correct drifts of more than 1‰ not accounted for by the internally calibrated data. A 3‐point calibration with four samples bracketed by two sets of standards provides the highest precision and accuracy, that is, the highest reproducibility for our carbonate samples. The third standard stabilizes the calibration curve and enables to better identify individual outliers.

**Conclusions:**

Our standard‐bracketing method enables accurate and precise (< 0.1‰) measurements of δ^13^C and δ^18^O values of calcite and DIC using the Delta Ray IRIS over a large range of values (ranging from approximately −37‰ to +2‰ in δ^13^C and from approximately −25‰ to −2‰ in δ^18^O).

## 1 Introduction

The measurement of stable carbon and oxygen isotopes provides valuable insights into numerous biological or geological processes, such as plant respiration [1], volcanic activity [2], or past climate or environmental variability [3], to name just a few. Over the past decades, numerous methods for analysis of δ^13^C and δ^18^O values, such as isotope ratio mass spectrometry (IRMS) [4] or cavity ring‐down spectroscopy [5], have been developed. The Thermo Scientific Delta Ray Series Isotope Ratio Infrared Spectrometer (IRIS) with a universal reference interface, Uri Connect, is an alternative to conventional IRMS. Infrared spectrometers are less difficult to operate than most IRMS systems and are also suitable for field applications due to their smaller size and weight. Another advantage is their ability to simultaneously measure different isotope ratios without the need of chemical pretreatment. The basic principle of an IRIS is the observation of spectral features, which are directly assigned to the isotopic molecular species of interest. To obtain the spectral features, different optical techniques, such as cavity ring down spectroscopy, Fourier‐transform infrared spectroscopy, laser photoacoustic spectroscopy and direct absorption spectroscopy, can be used [6]. In case of direct absorption spectroscopy, infrared light is absorbed by exciting the rotational and vibrational energy state of a certain molecule [7]. The transitions in the rotational and vibrational energy state are characteristically different for molecular species that differ only in their isotopic composition, so called isotopologues [8].

The Delta Ray IRIS is able to continuously measure the concentration as well as the δ^13^C and δ^18^O values of CO_2_ in ambient air [9]. If coupled to the URI Connect, the instrument is also able to measure the concentration as well as the δ^13^C and δ^18^O values of discrete CO_2_ samples. This enables to investigate a variety of samples, ranging from gases, solvents or solids, such as carbonates. The instrument is operated via the Qtegra Intelligent Scientific Data Solution (ISDS) software, which enables the user to acquire data by definable workflows in a LabBook. Using the internal CO_2_ reference gas, the Qtegra software automatically performs an internal scale correction, which consists of three different calibration routines. First, a linearity calibration evaluates the relationship between the concentration of CO_2_ and the isotope ratios by applying an experimentally derived correction factor to the raw isotope ratios. Second, a delta scale calibration, which is based on a 2‐point calibration using two reference gas tanks connected to the instrument, is performed. Third, a 2‐point calibration for CO_2_ concentration is performed using the connected reference gases [7]. These determined relationships are assumed to be sufficiently constant. However, for analysis of discrete carbonate samples, the manufacturer recommends performing an additional correction with carbonate standards [10] and reports an accuracy of < 0.1‰ for the δ^13^C and δ^18^O values of carbonate samples [10] and a precision of 0.3‰ for the δ^13^C values of DIC samples [11].

Here, we show that measurements of discrete carbonate and DIC samples with the Delta Ray IRIS may be affected by an instrumental drift, which has a significant effect on the calibrated δ^13^C and δ^18^O values. Thus, the accuracy obtained by the suggested approach [10, 11] may be much lower. This is also consistent with findings from Flores et al. (2017) [12] who found a systematic bias in the measured isotope values during measurements of CO_2_ in ultra dry air using the default procedure of the Delta Ray. This was then corrected by applying a 2‐point calibration using two extra CO_2_‐in air reference gases, yielding a substantially increased precision [12]. Here, we developed a 3‐point calibration method and tested different standard‐bracketing techniques for their effectiveness to account for the instrumental drift observed for carbonate samples. We also developed an R code [13], which enables the user to automatically evaluate and calibrate the measured δ^13^C and δ^18^O values. The code provides a variety of options to adjust the bracketing parameters and correction methods.

## 2 Experimental

In order to test the precision and accuracy of the Delta Ray IRIS instrument, we analyzed a homogenous synthetic carbonate (“RothCarbonate”, RC) with known δ^13^C and δ^18^O values, two homogenous in‐house standards (“MaxMarble”, MM, and “VWRCarbonate”, VC), and a certified reference material (IAEA‐612). The δ^13^C and δ^18^O values of the in‐house standards and the synthetic carbonate were determined on a Delta V isotope‐ratio mass spectrometer (IRMS), coupled to a GasBench system at the Max Planck Institute for Chemistry, Mainz. RC and VC are commercially purchased, technically precipitated CaCO_3_ powders. MM originates from a slab of Carrara‐marble that was crushed and ground into a fine powder. IAEA‐612 is a certified reference material purchased from the International Atomic Energy Agency (IAEA) in Vienna, which was obtained from a commercially available carbonate chemical product [14]. The carbonate standards cover a wide range of δ^13^C and δ^18^O values on the VPDB scale and are therefore suitable for bracketing a variety of carbonate samples of different isotope composition. The δ^13^C values range from −36.72‰ to +2.14‰, and the δ^18^O values are between −25.35‰ and −1.73‰ (Table 1).

**TABLE 1:** δ^13^C and δ^18^O values of the three carbonate standards.

Sample name	δ^13^C (‰)	δ^18^O (‰)
MaxMarble	2.14 ± 0.04	−1.73 ± 0.04
VWRCarbonate	−9.41 ± 0.09	−25.35 ± 0.10
IAEA‐612^a^	−36.72 ± 0.02	−12.08 ± 0.06
RothCarbonate	−8.73 ± 0.08	−18.33 ± 0.10

^a^ Assonov et al. 2021 [14]; all values are given vs. VPDB.

For measurements of DIC samples, a solution was prepared by dissolving 10 mmol/L NaHCO_3_ in MQ‐Water, which was previously sparged of any dissolved CO_2_ by bubbling Ar through the water column for several minutes (for details on the preparation of the solution see, e.g., Hansen et al. 2017 [15]). The δ^13^C and δ^18^O values of the NaHCO_3_ powder were analyzed alongside the DIC samples in the experiments presented here.

### 2.1 Sample Preparation and of Stable Isotope Analysis

#### 2.1.1 Carbonate Samples

The sample handling followed the application note for measuring δ^13^C and δ^18^O values of solid carbonate samples with the Delta Ray IRIS and Uri Connect [10]. Discrete portions of the carbonate sample were weighed to 400–600 μg. The samples were subsequently stored in 12 mL Labco Exetainer vials and sealed with gas‐tight rubber septa (Labco Ltd., Lampeter, U.K.). Using the Teledyne CETAC ASX‐7100 auto‐sampling unit, the vials were flushed with CO_2_‐free air (ALPHAGAZ 1 Air) for 180 s. Subsequently, 10 droplets of phosphoric acid (> 99.9% H_3_PO_4_) were added to each vial for digestion of CaCO_3_ into CO_2_. The reaction time was 90 min at 72°C. Note, that these conditions are slightly different to those in the application note [10]. The reaction time for the first sample and temperature was adapted according to Spötl and Vennemann (2003) [4]. The amount of acid was also increased compared to the application note in order to ensure a complete coverage of the carbonate samples at the bottom of the Exetainer vials. Also note that the reaction time of 90 min is only valid for the first sample. As the autosampler system is not capable of acidifying the samples automatically, phosphoric acid was added manually using a syringe. Thus, the reaction times range from 90 min for the first sample up to approximately 24 h for the last sample. We consider the Exetainer septa to be properly sealed during this period of time. However, drifts caused by the different reaction times cannot be ruled out. Therefore, the standard bracketing method is of importance as each bracket of standards has a reaction time which is of similar magnitude as for the samples within the corresponding bracket. Isotope effects potentially caused by different reaction times can be accounted for this way.

#### 2.1.2 DIC Samples

For the DIC samples, approximately 1 mL of NaHCO_3_‐solution was injected into 12‐mL Exetainer vials previously flushed with carrier gas (CO_2_ free air, see above). Subsequently, the samples were acidified by adding phosphoric acid (> 99.9% H_3_PO_4_) to each vial for digestion of the dissolved carbon species into CO_2_. The reaction time was 24 h at room temperature (i.e., 22°C). The corresponding carbonate standards were treated analogously.

### 2.2 Measurement Routine

To determine the best number of carbonate samples between two bracketing sets of standards and to test for a potential effect of the sample mass, three measurement runs were performed. The first and the second run consisted of 59 samples in total, that is, 32 “unknown” samples and 27 carbonate standards. Eight blocks of four carbonate samples (RC) were analyzed between nine sets of the three standards (MM, VC, and IAEA‐612). The sample weight was between 400 and 600 μg. The third measurement run consisted of 32 unknown samples (RC) and 15 carbonate standards, with four blocks of eight carbonate samples (RC) between five sets of the three standards. The sample weight was again between 400 and 600 μg. The DIC samples were analyzed analogously to the carbonate samples and as described in Mandic et al. 2017 [11]. The measurement runs consisted of eight blocks of four DIC samples between nine sets of the three carbonate standards. For the DIC samples, we only performed one measurement run.

The measurement routine followed the application note for carbonate samples [10]. Every measurement run started with the cleaning procedure of the autosampler, where the Delta Ray Connect releases a stream of carrier gas through the autosampler and the sample dryer for 600 s [10]. The internal reference gas was measured between all samples and standards. Each standard gas had a flush time of 60 s and a measurement time of 180 s. The samples and carbonate standards were flushed for 40 s and measured for 180 s. After a measurement is completed, the results are saved in the corresponding LabBook. During the measurement, the Delta Ray Connect integrates the measured values over a time interval of 1 min. These values are listed for each sample, together with the statistical mean and 1SD over the total measurement time of 3 min, which gives the internal precision of the machine. The LabBook allows the user to reevaluate the measurement data without using the internal gas standards for calibration. In order to evaluate the effect of the internal calibration on the instrumental drift, we evaluated all measurement runs with and without using the reference gas standard. The results can be exported to an Excel spreadsheet. For the data evaluation using R, the user needs to manually convert the Excel spreadsheet to a CSV UTF‐8 document.

### 2.3 Data Correction

In the following, we will discuss how the isotope data as obtained from the LabBook were evaluated. First, the δ^18^O values calculated by the Qtegra software, which are reported relative to the VPDB‐CO2 standard, need to be corrected for acid fractionation. This is done using the fractionation factor $\alpha_{{\mathrm{CO}}_{2}\left(\text{ACID}\right)-\text{calcite}}$ reported by Kim et al. 2015 [16]. The δ^18^O values are then recalculated vs. the VPDB standard to provide data conformity (Equation (1)). The isotope ratios,^18^R, of the standards used in this study are listed in Table 2.

(1) δ18OxVPDBacid−corrected=δ18Ox,VPDB−CO21000+1×R18VPDB−CO2αCO2ACID−calciteR18VPDB−1×1000

**TABLE 2:** Isotope ratios of the standards used.

Standard	^13^C/^12^C	^18^O/^16^O
VPDB	0.011113^a^	0.0020672^b^
VPDB‐CO_2_	0.0112372^c^	0.00208839^a^

^a^ Camin et al. 2025 [17].

^b^ Mook 2000 [18].

^c^ Gonfiantini et al. 1995 [19].

#### 2.3.1 The 2‐Point Calibration

For the 2‐point data calibration, MM and VC were used as standards. The third standard, IAEA‐612, was also analyzed, but not taken into account. To generate a calibration curve, the measured δ^13^C and δ^18^O values of all standards are averaged and expressed as raw isotope ratios using the following equation:

(2) $$R_{x}=\left(\frac{\delta_{x}}{1000}+1\right)\times R_{\mathrm{std}}$$

These isotope ratios are then plotted against the expected carbon and oxygen ratios (Table 3). Linear regression of these data points then results in an average calibration function based on all standards of the following form:

(3) $$R_{\text{expected}}=\text{slope}\times R_{\text{measured}}+y\;\text{intercept}$$

**TABLE 3:** Expected isotope ratios for the standards.

Standard name	^13^C/^12^C	^18^O/^16^O
MaxMarble	0.01113678182	0.00206362374
VWRCarbonate	0.01100842667	0.00201479648
IAEA‐612	0.01070493064	0.00204222822

The corresponding calibration functions are then used to correct the measured carbon and oxygen isotope ratios of all samples [10]. Finally, the corrected isotope ratios are converted into delta values.

#### 2.3.2 The 3‐Point Calibration

For the 3‐point data calibration, we evaluated IAEA‐612 in addition to MM and VC. This stabilizes the calibration curve by adding a third data point and, in particular, by extending the range towards lower ^13^C/^12^C isotope ratios. The calibration curve is again established by linear regression. The resulting average calibration functions based on all standards (Equation (3)) are subsequently used to correct the measured carbon and oxygen isotope ratios of all samples, which are then converted into delta values.

#### 2.3.3 Standard Bracketing

With the standard‐bracketing technique, every block of samples is calibrated by the two bracketing standard measurements. Thus, instead of averaging all measured isotope ratios of the standards and applying linear regression (see above), the regression is now only performed for the two bracketing standard measurements. For the measurement of eight blocks of samples (first run), this results in eight individual calibration curves. In addition, rather than averaging the individual isotope ratios for each bracket, the linear fit is performed with the individual isotope ratios. We performed a weighted linear fit taking into account the uncertainty of the standard measurements as well as Gaussian error propagation of the uncertainty of the fitting parameters to obtain the corrected isotope values. All isotope ratios are subsequently converted into delta values using Equation (2). The corresponding uncertainties were calculated using error propagation.

### 2.4 Data Evaluation Using R

In order to evaluate the generated stable isotope data, we developed an R code automatically correcting the measurements using the different carbonate standards. The code was developed using version 4.3.1 (“Beagle Scouts”) of the R language for statistical computing [13] and uses the packages “Hmisc” [17], “tcltk” [13], “openxlsx” [18], and “extrafont” [19]. “Hmisc” is used for plotting error bars to the individual values, “tcltk” is used for the identifier tool to remove potential outliers, “openxlsx” is used for the export of the evaluated data into an excel result sheet, and “extrafont” is used for the correct display of special characters in the plots. The user only needs to define a few parameters, such as the total number of samples and the width of the brackets (i.e., the number of samples per bracket). The code automatically identifies samples with too low CO_2_ content and provides options to handle missing standard values and outliers. In case of missing standard values in the results sheet, the code linearly interpolates by averaging the two surrounding standard measurements. If the missing standard value occurs in the first (last) set of standards, the value of the following (previous) standard measurement is used. The code including a more detailed instruction is provided in the supplement.

## 3 Results

In the following, we present the results of the different correction methods for the first measurement run to generally illustrate and explain the correction process. A summary of the results of the other experiments and the corresponding figures is provided in the Supporting Information.

### 3.1 Uncorrected Data

**FIGURE 1:**
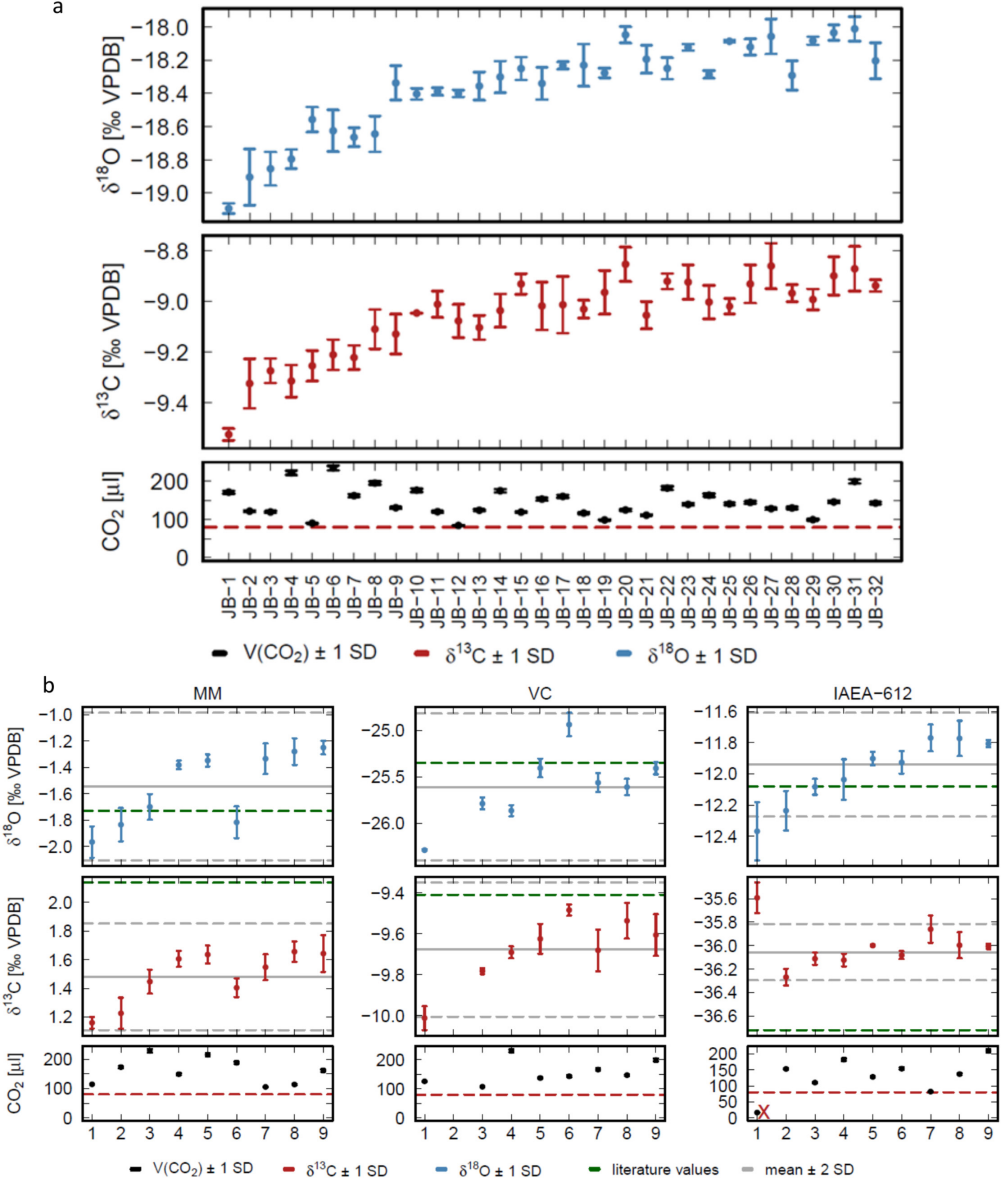
Results of the first measurement run, internally calibrated against the internal reference gas standard. (a) δ^18^O and δ^13^C values of the RC samples; (b) δ^18^O and δ^13^C values of the carbonate standards. The red x indicates outliers.

The results of the δ^13^C and δ^18^O measurements of the first measurement run of the carbonate samples are shown in Figure 1a; the results of the corresponding carbonate standards, calibrated against the internal reference gas standard, are shown in Figure 1b. Over the whole run, the δ^13^C and δ^18^O values of both the standards and the samples show a significant increase by up to +1‰. The Qtegra software allows manually discarding gas standards from a measured LabBook and recalculating the measured isotope values. In order to obtain delta values, at least one gas standard is required. Interestingly, when excluding all gas standards, except for one, we observe a drift towards more negative values similar with a similar magnitude (Figure S1).

The results of the second measurement run, calibrated against the internal reference gas standard, are shown in Figure S2. Figure S2b shows the δ^13^C and δ^18^O values of the carbonate standards; the results of the RC samples are shown in Figure S2a. Over the whole run, we observe a drift of about +0.7‰ towards more positive δ^13^C and δ^18^O values. Without using the internal reference gas standards, the drift is on the same order of magnitude (Figure S3).

It is obvious that some of the measured standard values are significantly different than the expected values (visible, e.g., in Figure 1b, MM standard 6 and in IAEA‐612 standard 1, as well as in Figures S1–S3). These values are considered as outliers and are marked with a red x. For some of these values, this can be explained by low CO_2_ concentrations (i.e., < 80 μL total CO_2_ concentration, e.g., Figure 1b, panel IAEA, first value). Other outliers may be due to leaking septa or exetainer caps, which are not perfectly tight. For further evaluation, the outliers were excluded and replaced by values obtained by interpolating (averaging) the surrounding two standard measurements.

### 3.2 The 2‐ and 3‐Point Calibrations

**FIGURE 2:**
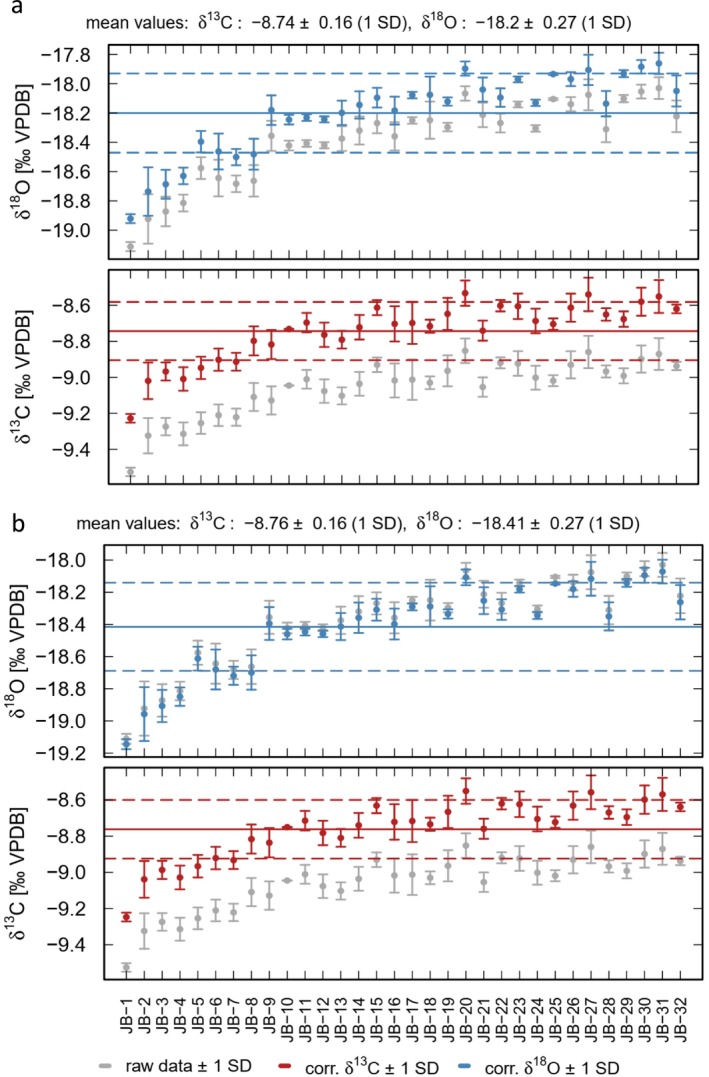
Corrected δ^13^C (red) and δ^18^O (blue) values of the RC samples from the first measurement run. Gray symbols indicate the uncorrected data. The results were calibrated applying a 2‐point calibration using (a) MM and VC and (b) MM and IAEA‐612.

Figure 2 shows the results for the 2‐point calibration as suggested by Smajgel et al. 2018 [10]. Using the MM and VC standards, the first run yields average δ^13^C and δ^18^O values of −8.74‰ and −18.2‰, respectively. The correction shifts both the δ^13^C and δ^18^O values towards more positive values (Figure 2a). The precision (1SD) is ± 0.16‰ for the δ^13^C values and ± 0.27‰ for the δ^18^O values, respectively (Figure 2a).

For our dataset, three combinations of standards are possible for a 2‐point calibration (MM and VC, MM and IAEA‐612 as well as VC and IAEA‐612). The corrections using MM and VC and VC and IAEA‐612 provided the same results. Using MM and IAEA‐612, the values of the RC carbonate sample are slightly more negative, whereas the precision stays the same (i.e., δ^13^C = −8.76‰ ± 0.16‰ and δ^18^O = −18.41‰ ± 0.27‰; Figure 2b). For this combination of standards, the correction shifts all δ^13^C values towards more positive values, whereas the δ^18^O values are shifted towards slightly more negative values (Figure 2b). This highlights the importance of appropriate standard materials with a range of isotope ratios completely covering the expected values of the samples. However, due to the design of the correction, which is based on an average correction factor for the whole measurement run, the observed drift within the RC sample measurements (Figure 1) remains the same (Figure 2). Thus, the 2‐point calibration is not sufficient to correct for the observed drift in the measured isotope values.

Applying the 2‐point calibration to the first measurement run without using the internal gas standards (Figure S1), the corresponding mean‐corrected values are δ^13^C = −8.77‰ ± 0.09‰ and δ^18^O = −18.3‰ ± 0.2‰ for MM and VC (Figure S4a), δ^13^C = −8.75‰ ± 0.09‰ and δ^18^O = −18.2‰ ± 0.19‰ for MM and IAEA‐612 (Figure S4b) as well as δ^13^C = −8.74‰ ± 0.09‰ and δ^18^O = −18.24‰ ± 0.19‰ for VC and IAEA‐612 (Figure S4c). With the 3‐point calibration and including the internal gas standards, the mean‐corrected δ^13^C value is −8.77‰ ± 0.09‰ and the mean‐corrected δ^18^O value is −18.24‰ ± 0.27‰ (Figure S5a). Without including the internal gas standards, the mean‐corrected δ^13^C value is −8.76‰ ± 0.09‰ and the mean‐corrected δ^18^O value −18.22‰ ± 0.19‰, respectively (Figure S5b).

For the second run, we obtain mean‐corrected δ^13^C values between −8.77‰ and −8.78‰ and mean‐corrected δ^18^O values between −18.05‰ and −18.18‰ using a 2‐point calibration and all combinations of carbonate standards as well as a 3‐point calibration. The correction shifts the δ^13^C values by about +0.4‰ and the δ^18^O values by about +0.9‰ towards more positive values (Figure S6). The precision is ±0.10‰ for the δ^13^C values and ±0.19‰ for the δ^18^O values, respectively. For the same run but without internal gas standards, we observe mean‐corrected δ^13^C values between −8.76‰ and −8.77‰ and mean‐corrected δ^18^O values between −18.07‰ and −18.16‰ for all combinations of carbonate standards. The correction shifts the δ^13^C values by about +0.4‰ and the δ^18^O values by about +0.5‰ towards more positive values (Figure S7). The precision is ±0.06‰ for the δ^13^C values and ±0.12‰ for the δ^18^O values, respectively.

### 3.3 Standard Bracketing

**FIGURE 3:**
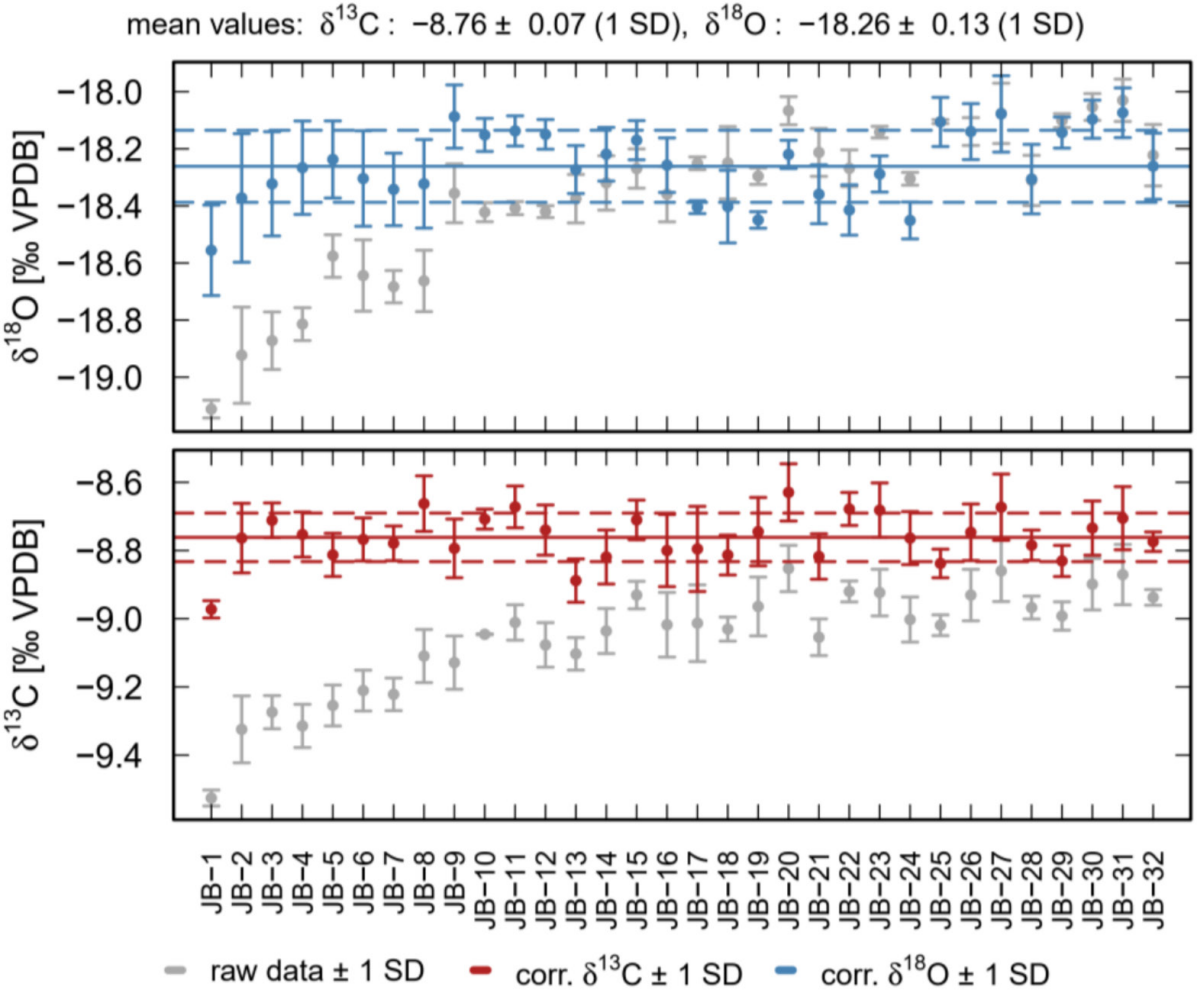
Corrected δ^13^C (red symbols) and δ^18^O (blue symbols) values of the RC samples from the first measurement run. Gray symbols indicate the uncorrected data. The values are calibrated using MM, VC, and IAEA‐612 and the standard bracketing technique.

Figures 2 and S4–S7 clearly show that the 2‐point calibration suggested by Smajgl et al. 2018 [10] is not sufficient to correct for the instrumental drift during a measurement run, which was up to +0.6‰ for the δ^13^C values and +1‰ for the δ^18^O values in our experiments. Applying a 3‐point calibration is also not sufficient to correct for the observed drift (Figure S5). Figure 3 shows the results obtained with the standard‐bracketing technique, which was developed to account for such drifts. The mean‐corrected δ^13^C value for the first measurement run (four samples between each bracket) is −8.76‰; the mean‐corrected δ^18^O value is −18.26‰ (Figure 3). It is obvious that the corrected delta values show a substantially lower drift that the uncorrected data, resulting in a precision of ±0.07‰ for the δ^13^C values and ±0.13‰ for the δ^18^O values (Figure 3). Without using the internal gas standards, the results are even more precise with δ^13^C = −8.76 ± 0.06 and δ^18^O = −18.14‰ ± 0.12‰ (Figure S8). Note the slight difference between the individual blocks of samples.

In order to test the performance of the bracketing technique, we re‐evaluated the first measurement run removing every second block of standards to obtain a data set with eight samples between each standard block. This results in a mean‐corrected δ^13^C value of −8.77‰ ± 0.08‰ and a mean‐corrected δ^18^O value of −18.29‰ ± 0.12‰, respectively (Figure S9a). The offset between the different blocks of samples is still visible, especially for the first block. This suggests that the bracketing is not tight enough to correct for the observed drift. However, the uncertainty is almost identical as for the narrower bracketing. Without the internal gas standards, the mean‐corrected values are −8.76‰ ± 0.06‰ for δ^13^C and −18.23‰ ± 0.12‰ for δ^18^O. In this case, no significant differences between the individual blocks are visible (Figure S9b). In summary, there are no significant differences between the narrower and the wider bracketing (four vs. eight samples per bracket).

### 3.4 The Advantage of Carbonate Standard Bracketing

**FIGURE 4:**
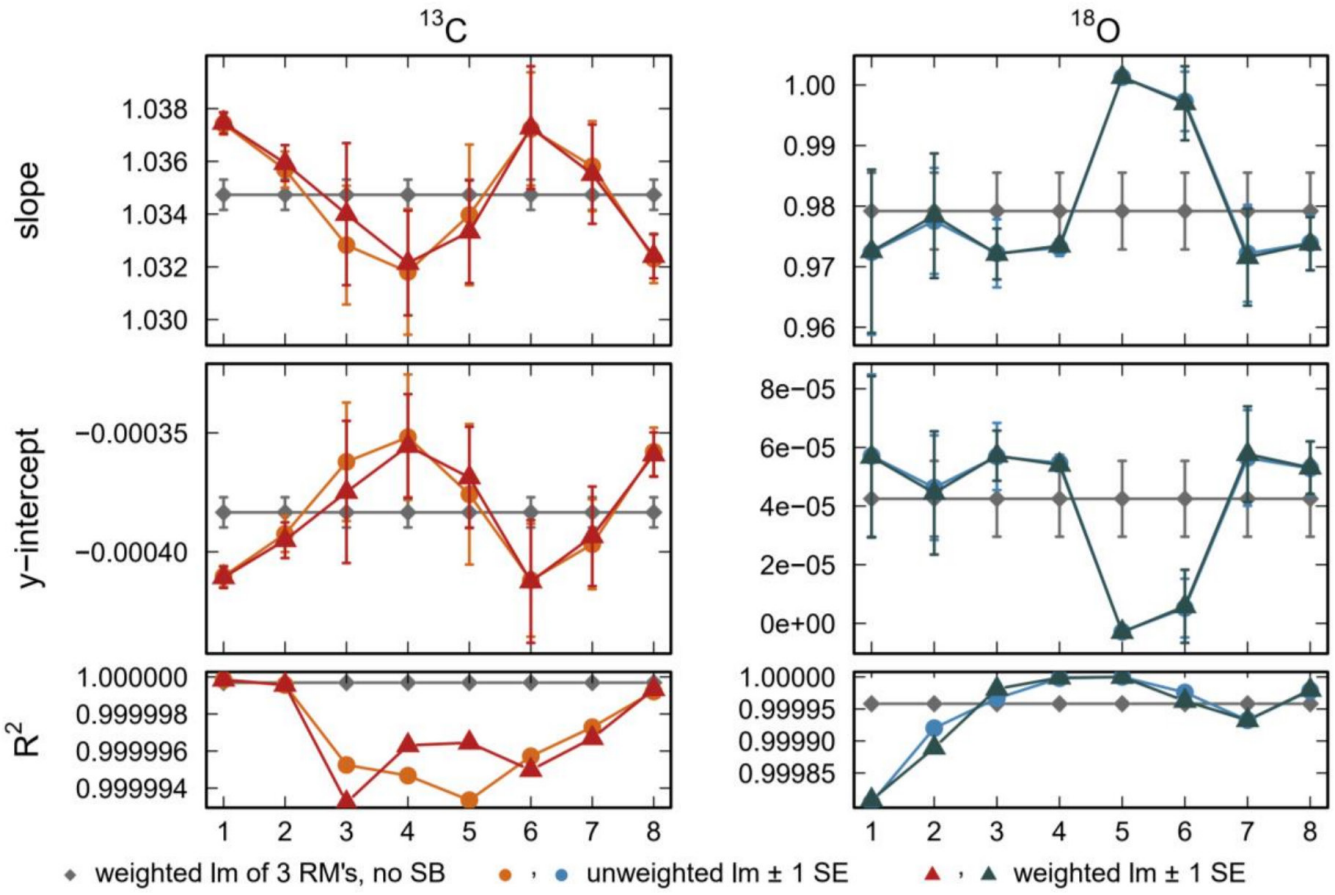
Linear regression coefficients for the first measurement run using the internal gas standard. The colored symbols show the regression coefficients for the individual standard blocks obtained from the standard bracketing technique (triangles show the weighted regression, circles the unweighted regression). The gray diamonds show the average coefficient obtained by the 3‐point calibration.

In order to discuss the differences between the 2‐ and 3‐point calibrations and the standard bracketing technique, we compare the linear regression coefficients obtained from both calibration methods. The 2‐ and 3‐point calibrations are based on a single fit applied to the mean values of the measurements of the standard and, thus, yield a single pair of correction coefficients for the whole data set. Consequently, the calibration does not correct for potential drifts in both the standard and sample measurements (Figures 1, 2, 3). In contrast, for the standard bracketing method, individual correction coefficients are determined for every sample block. Consequently, the evolution of the correction coefficients obtained from the measurements of the standards accounts for the drift of the sample measurements. This allows to quantify and to correct for the drift resulting in a higher precision of the sample measurements (± 0.07‰ vs. ± 0.16‰ for δ^13^C and ± 0.13‰ vs. ± 0.27‰ for δ^18^O in the first measurement run, compare Figures 2 and 3). This is also obvious from Figure 4 showing the correction coefficients for the first run. We calculated the regression coefficients both with and without error weighting (triangles and circles in Figure 4). Over the run, the regression coefficients show significant deviations from the mean coefficient used for the 2‐ and 3‐point calibrations (RM in Figure 4). The correction coefficients show no systematic deviation from the mean coefficient over the run. This highlights the importance of an individual correction for each standard bracket in order to obtain the most accurate (corrected) results. Without using the internal gas standards, the results are similar (Figure S10). Interestingly, the deviations from the mean coefficient seem to be smaller in this case, even if they are still significant. The correction coefficients for the second measurement run and for the dataset with eight samples between each bracket, both with and without using the internal gas standards, are shown in Figures S11 and S12. They all show a similar behavior, again highlighting the importance of the standard bracketing.

**FIGURE 5:**
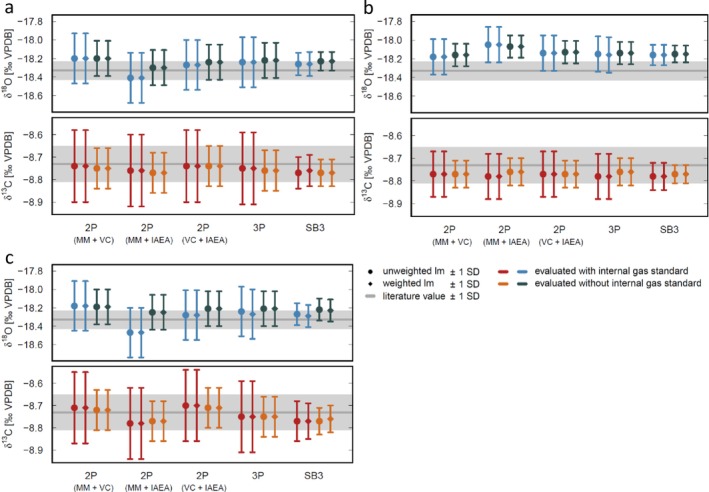
Comparison of the corrected values obtained by the different correction methods ((a) first measurement run; (b) second run; (c) first run with eight samples per bracket). 2P and 3P refers to the overall mean fit (i.e., 2‐ and 3‐point calibrations); SB refers to standard bracketing. Different colors indicate the evaluation with and without the internal gas standards.

Figure 5 shows a comparison of the mean‐corrected values for all correction approaches. All values are in agreement with the corresponding expected δ^13^C and δ^18^O values for RC (gray shaded area in Figure 5, compare Table 1). The smallest uncertainties and, thus, the highest reproducibility are obtained by the standard bracketing method. Interestingly, the precision is also always substantially better without using the internal gas standards (dark blue and orange diamonds in Figure 5). The narrower standard bracketing (four samples per bracket) does not show a significantly better precision than the wider bracketing (eights samples per bracket). Nevertheless, the narrower bracketing may be better suited to account for rapid changes during a measurement run.

## 4 Results of the DIC Samples

**FIGURE 6:**
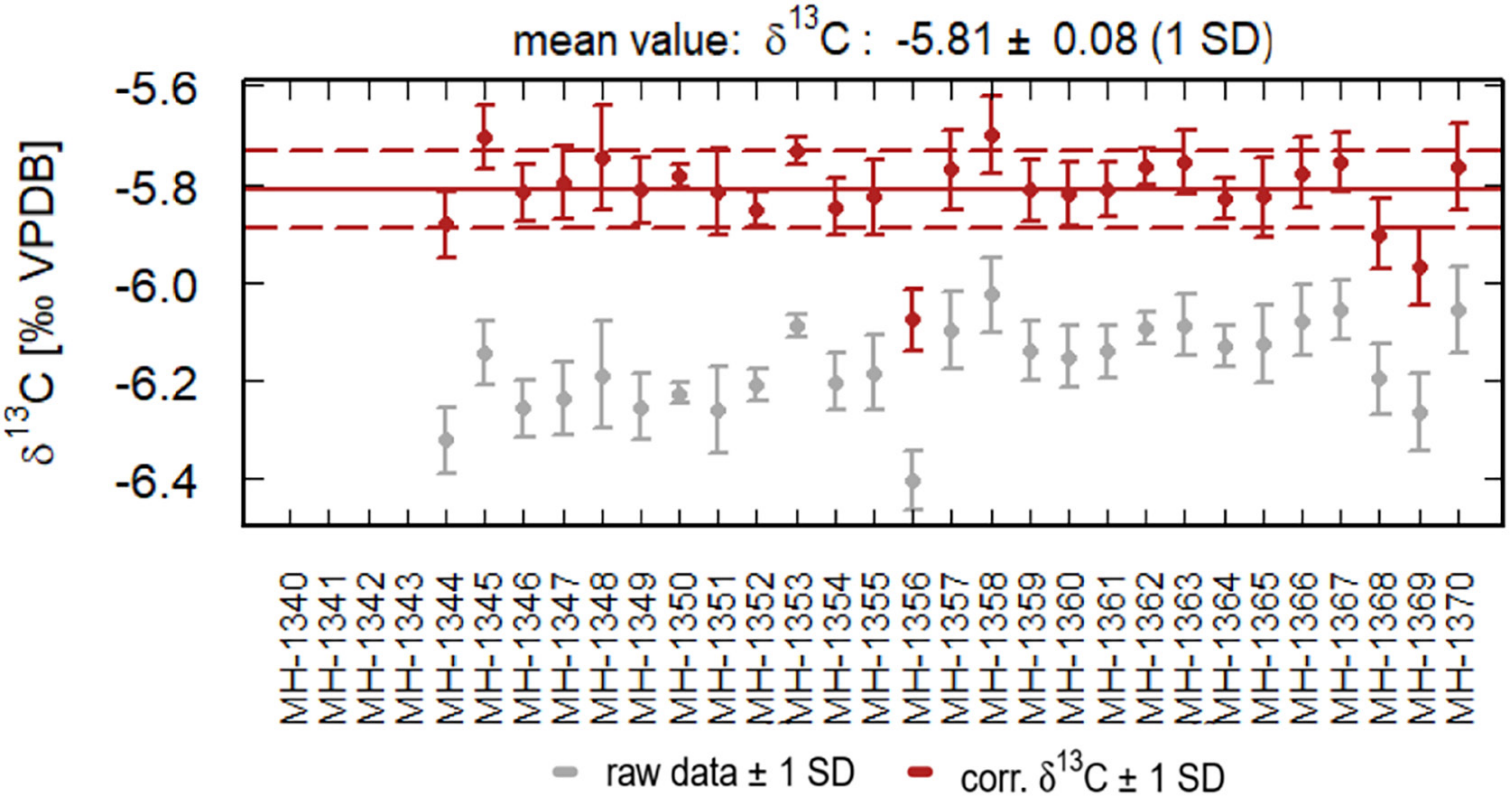
Corrected δ^13^C values of the DIC samples. The red line shows the mean value; the dashed lines the 1SD range.

The same procedure can be applied to δ^13^C values of DIC measured by the Delta Ray. As for the carbonate samples, the DIC data were corrected using three standards, the standard bracketing approach and four samples per bracket. The mean δ^13^C value is −5.81‰ ± 0.08‰ (Figure 6). The first four δ^13^C values (MH‐1340‐MH1343, Figure 6) were excluded since they refer to the NaHCO_3_ powder used to prepare the solutions. The corresponding δ^13^C values are in agreement with the corrected δ^13^C values of the DIC samples (δ^13^C = −5.8 ± 0.07). Application of the standard bracketing technique again yields a substantially better precision than the 2‐point calibration (i.e., ± 0.08‰ vs. ± 0.8‰) proposed in the application note [11].

## 5 Conclusions

We demonstrated that δ^13^C and δ^18^O values of both carbonate and DIC samples determined using the Thermo Scientific Delta Ray Series IRIS with Uri Connect need to be corrected using a standard bracketing approach when aiming for the highest precision and accuracy. The data correction suggested by the manufacturer does not account for the observed instrumental drift in our measurements and is, thus, not sufficient. The application of the internal gas standards does not account for the instrumental drift and even appears to overcorrect the effect. Measuring four samples per bracket does not provide significantly better results than using a wider bracket (eight samples per bracket) but may still be useful in case of rapidly drifting conditions.

The developed R code allows automated and fast calibration of the measured stable isotope data. In addition, it enables to identify potential outliers and provides the opportunity to replace them by interpolated values.

## Author Contributions

**Maximilian Hansen:** conceptualization, methodology, investigation, funding acquisition, writing – original draft, data curation, project administration, supervision, formal analysis, validation, visualization, software. **Jakob Brettschneider:** methodology, software, data curation, investigation, writing – review and editing, visualization, validation, formal analysis, conceptualization. **Hubert Vonhof:** methodology, validation, investigation, data curation, formal analysis, writing – review and editing. **Denis Scholz:** supervision, conceptualization, methodology, investigation, software, writing – review and editing, validation, formal analysis, data curation.

## Conflicts of Interest

The authors declare no conflicts of interest.

## Supporting information


**Figure S1:** Results of the first measurement run, without internal gas standards. (a) δ^18^O and δ^13^C values of the RothCarbonate samples; (b) δ^18^O and δ^13^C values of the carbonate standards. The red x indicates an outlier, which is defined in the main text.
**Figure S2:** Results of the second measurement run, internally calibrated against the reference gas standard. (a) δ^18^O and δ^13^C values of the RC samples; (b) δ^18^O and δ^13^C values of the carbonate standards. The red x's indicate outliers which are defined in the main text.
**Figure S3:** Results of the second measurement run, without internal reference gas standards. (a) δ^18^O and δ^13^C values of the RothCarbonate samples; (b) δ^18^O and δ^13^C values of the carbonate standards samples. The red x's indicate outliers which are defined in the main text.
**Figure S4:** Corrected δ^13^C (red) and δ^18^O (blue) values of the RC samples of the first measurement run without internal reference gas standards. Gray symbols indicate the uncorrected data. The results were calibrated applying a 2‐point calibration with an overall mean fit through all standards using the (a) MM and VC, (b) MM and IAEA‐612, and (c) VC and IAEA‐612 carbonate standards.
**Figure S5:** Corrected δ^13^C (red) and δ^18^O (blue) values of the RC samples of the first measurement run (a) with and (b) without internal reference gas standards. Gray symbols indicate the uncorrected data. The results were calibrated applying a 3‐point calibration with an overall mean fit through all measured standards using the MM and VC and IAEA‐612 carbonate standards.
**Figure S6:** Corrected δ^13^C (red) and δ^18^O (blue) values of the RC samples of the second measurement run. Gray symbols indicate the uncorrected data. The results were calibrated applying a 2‐point calibration with an overall mean fit through (a) MM and IAEA‐612, (b) MM and VC, (c) VC and IAEA‐612, and (d) an overall mean fit through all three carbonate standards.
**Figure S7:** Corrected δ^13^C (red) and δ^18^O (blue) values of the RC samples of the second measurement run without internal reference gas standards. Gray symbols indicate the uncorrected data. The results were calibrated applying a 2‐point calibration with an overall mean fit through (a) MM and IAEA‐612, (b) MM and VC, (c) VC and IAEA‐612, and (d) an overall mean fit through all three carbonate standards.
**Figure S8:** Corrected δ^13^C (red) and δ^18^O (blue) values of the RC samples of the first measurement run without internal reference gas standards. Gray symbols indicate the uncorrected data. The values are calibrated against MM, VC and IAEA‐612 by applying a standard bracketing.
**Figure S9:** Corrected δ^13^C (red symbols) and δ^18^O (blue symbols) values of the RC samples of the first measurement run but using a wider bracket of 8 samples between two sets of standards: (a) with internal reference gas standards (b) without gas standards. Gray symbols indicate the uncorrected data. The values are calibrated against MM, VC, and IAEA‐612 by applying a standard bracketing.
**Figure S10:** Linear regression coefficients for the first measurement run, without reference gas standards. The colored symbols indicate the individual correction coefficients for every individual standard block obtained from the standard bracketing (triangles weighted lm, circles unweighted lm); the gray diamonds indicate the coefficients of the overall mean fit of the three reference materials.
**Figure S11:** Linear regression coefficients for the second measurement run: (a) internaly calibrated against the reference gas standards and (b) without internal reference gas standards. The colored symbols show the regression coefficients for the individual standard blocks obtained from the standard bracketing technique (triangles show the weighted regression, circles the unweighted regression). The gray diamonds show the average coefficient obtained by the 3‐point calibration.
**Figure S12:** Linear regression coefficients for the first measurement run but with a wider bracket width of eight samples between two blocks of carbonate standards, (a) internaly calibrated against the reference gas standards and (b) without internal reference gas standards. The colored symbols show the regression coefficients for the individual standard blocks obtained from the standard bracketing technique (triangles show the weighted regression, circles the unweighted regression). The gray diamonds show the average coefficient obtained by the 3‐point calibration.

**Data S1:** Supporting Information.

**Data S2:** Supporting Information.

**Data S3:** Supporting Information.

## Data Availability

The data that support the findings of this study are available from the corresponding author upon reasonable request.
