# Supplementary material for: Stable Carbon and Oxygen Isotope Analysis of Carbonates and DIC Using the Delta Ray Isotope Ratio Infrared Spectrometer (IRIS): Precise and Accurate Measurements Applying a 3‐Point Calibration and Standard Bracketing
Source: Rapid Commun Mass Spectrom. 2025 Dec 29;40(6):e70021. doi: 10.1002/rcm.70021 (PMC12748363; doi:10.1002/rcm.70021)
Supplement: Supplementary file 1 — Figure S1: Results of the first measurement run, without internal gas standards. (a) δ18O and δ13C values of the RothCarbonate samples; (b) δ18O and δ13C values of the carbonate standards. The red x indicates an outlier, which is defined in the main text. Figure S2: Results of the second measurement run, internally calibrated against the reference gas standard. (a) δ18O and δ13C values of the RC samples; (b) δ18O and δ13C values of the carbonate standards. The red x's indicate outliers which are defined in the main text. Figure S3: Results of the second measurement run, without internal reference gas standards. (a) δ18O and δ13C values of the RothCarbonate samples; (b) δ18O and δ13C values of the carbonate standards samples. The red x's indicate outliers which are defined in the main text. Figure S4: Corrected δ13C (red) and δ18O (blue) values of the RC samples of the first measurement run without internal reference gas standards. Gray symbols indicate the uncorrected data. The results were calibrated applying a 2‐point calibration with an overall mean fit through all standards using the (a) MM and VC, (b) MM and IAEA‐612, and (c) VC and IAEA‐612 carbonate standards. Figure S5: Corrected δ13C (red) and δ18O (blue) values of the RC samples of the first measurement run (a) with and (b) without internal reference gas standards. Gray symbols indicate the uncorrected data. The results were calibrated applying a 3‐point calibration with an overall mean fit through all measured standards using the MM and VC and IAEA‐612 carbonate standards. Figure S6: Corrected δ13C (red) and δ18O (blue) values of the RC samples of the second measurement run. Gray symbols indicate the uncorrected data. The results were calibrated applying a 2‐point calibration with an overall mean fit through (a) MM and IAEA‐612, (b) MM and VC, (c) VC and IAEA‐612, and (d) an overall mean fit through all three carbonate standards. Figure S7: Corrected δ13C (red) and δ18O (blue) values of the RC samples of [file RCM-40-e70021-s002.docx]

**Supplementary material to: Stable carbon and oxygen isotope analysis of carbonates and DIC using the DeltaRay^TM^ Isotope Ratio Infrared Spectrometer (IRIS): precise and accurate measurements applying a three-point calibration and standard bracketing by M. Hansen et al.**

a)


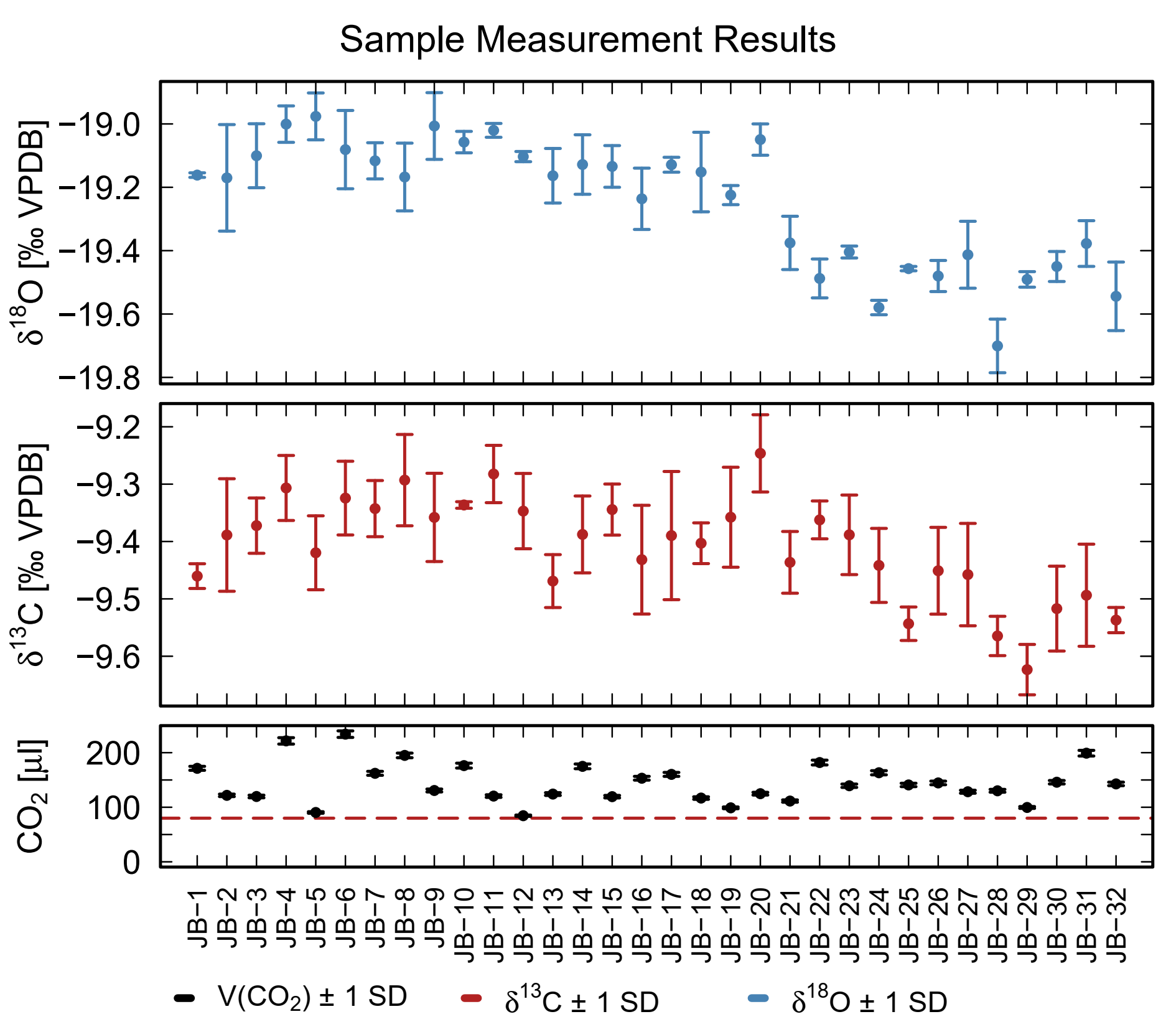

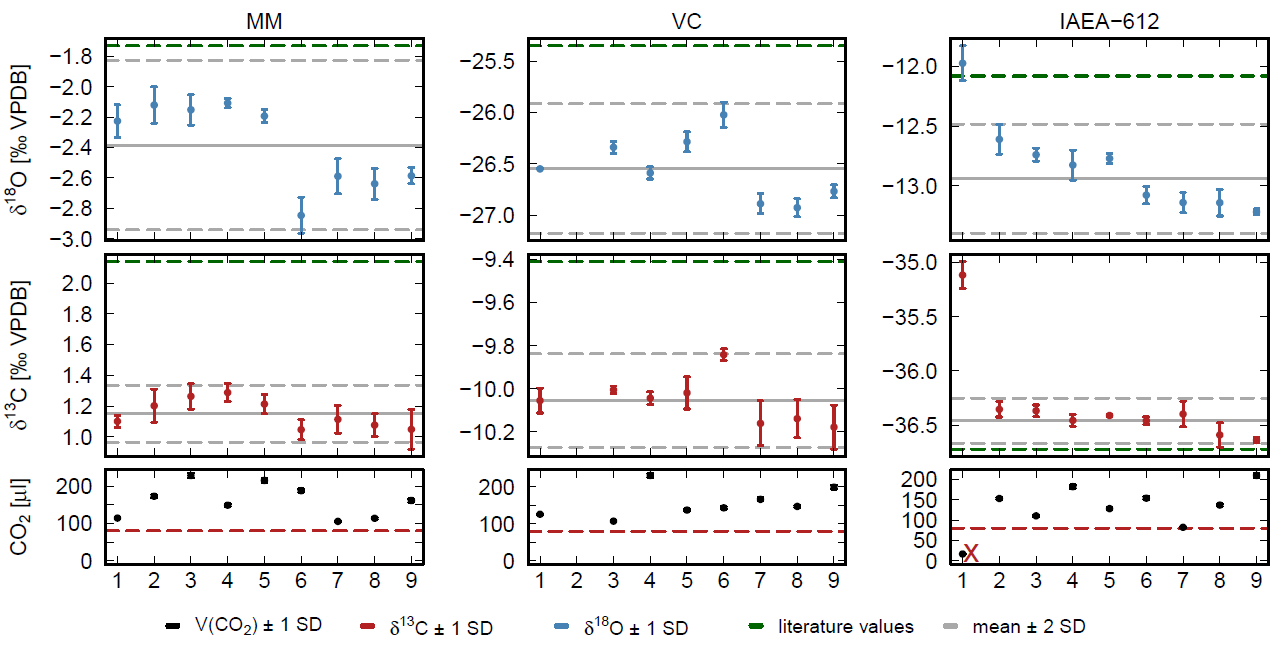


b)

**Figure S1:** Results of the first measurement run, without internal gas standards. a) δ^18^O and δ^13^C values of the RothCarbonate samples; b) δ^18^O and δ^13^C values of the carbonate standards. The red x indicates an outlier, which is defined in the main text.


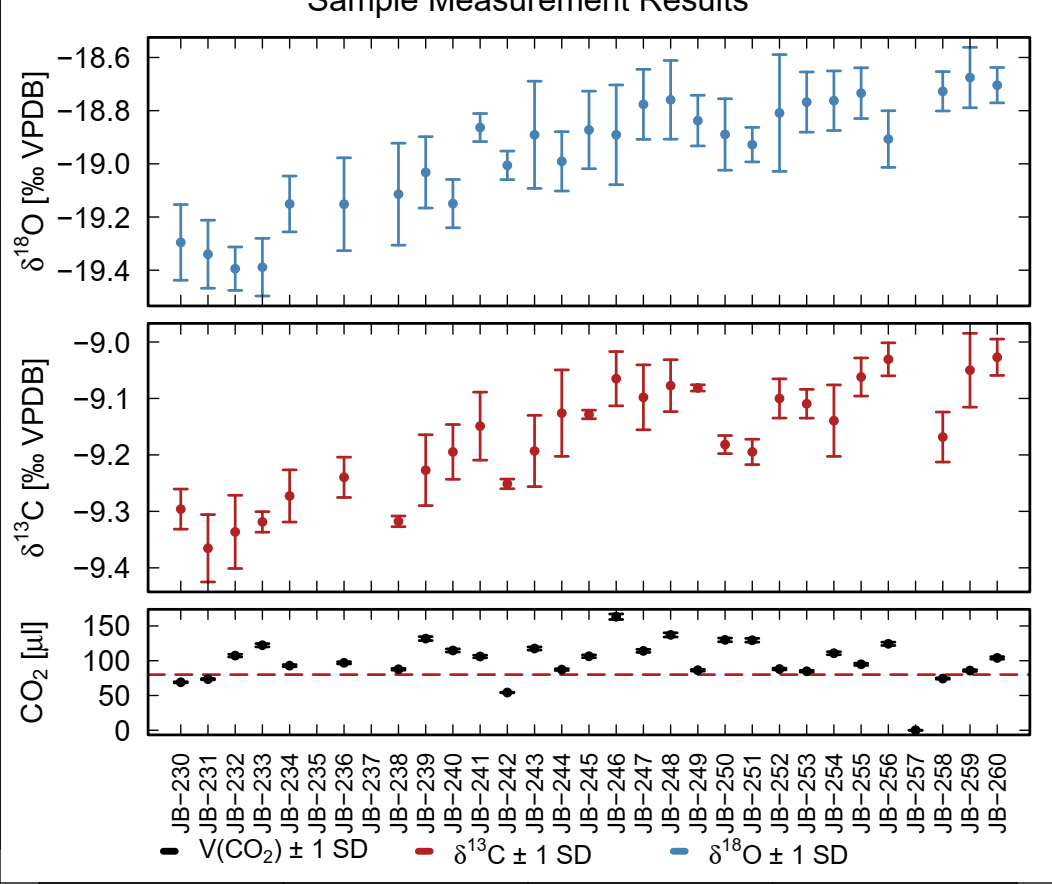


b)

a)


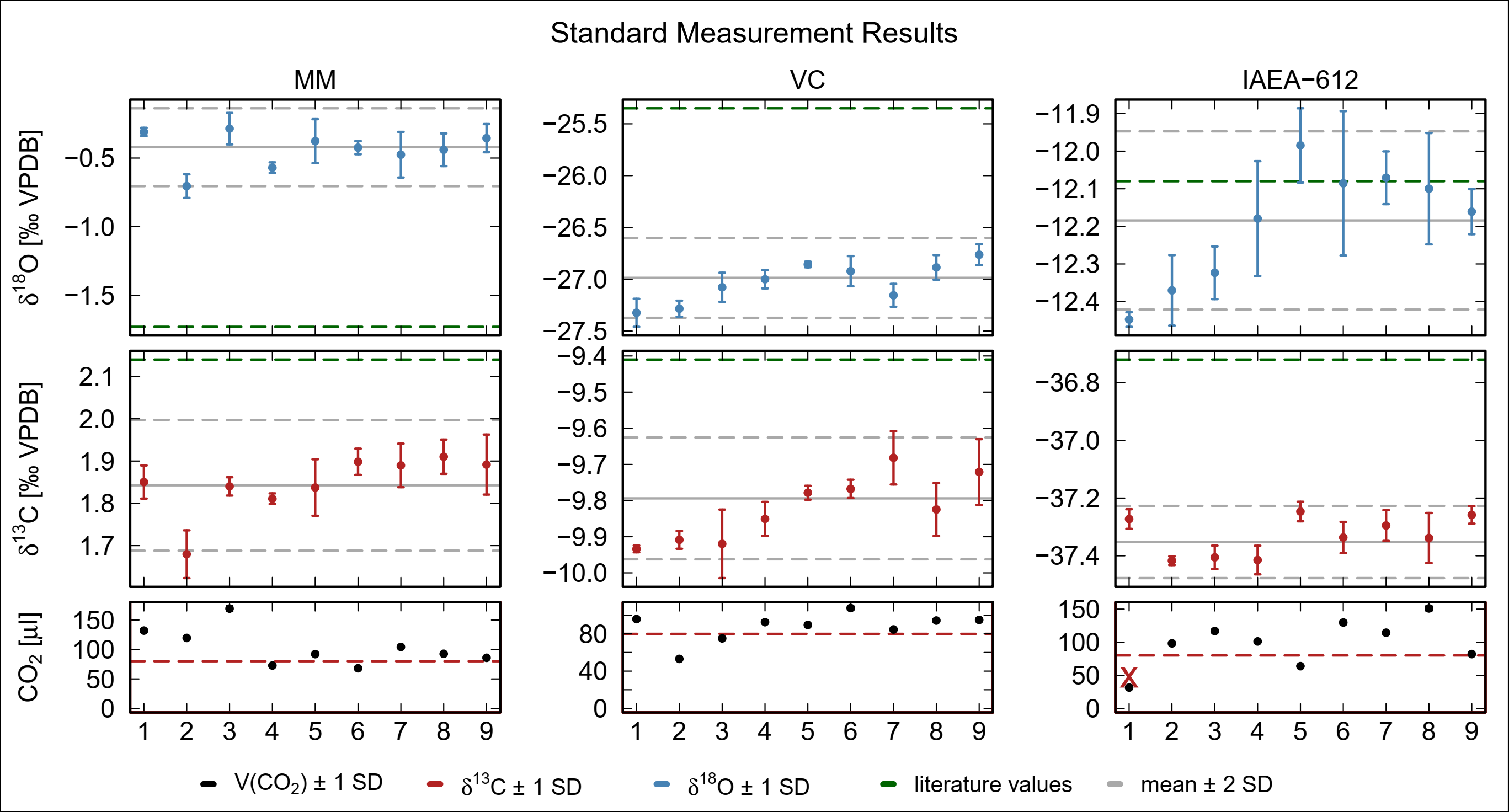


**Figure S2:** Results of the second measurement run, internally calibrated against the reference gas standard. a) δ^18^O and δ^13^C values of the RC samples; b) δ^18^O and δ^13^C values of the carbonate standards. The red x’s indicate outliers which are defined in the main text.


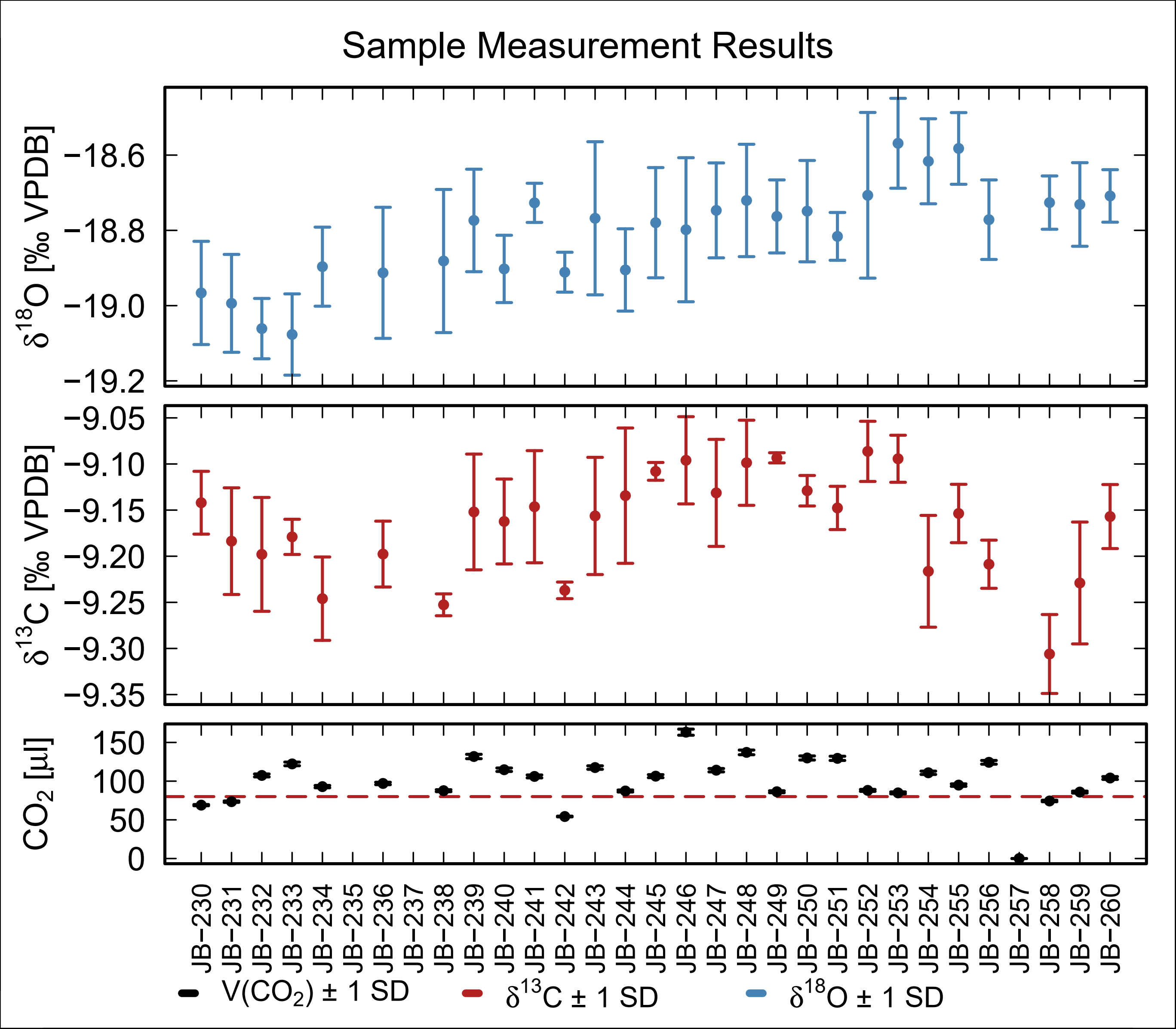


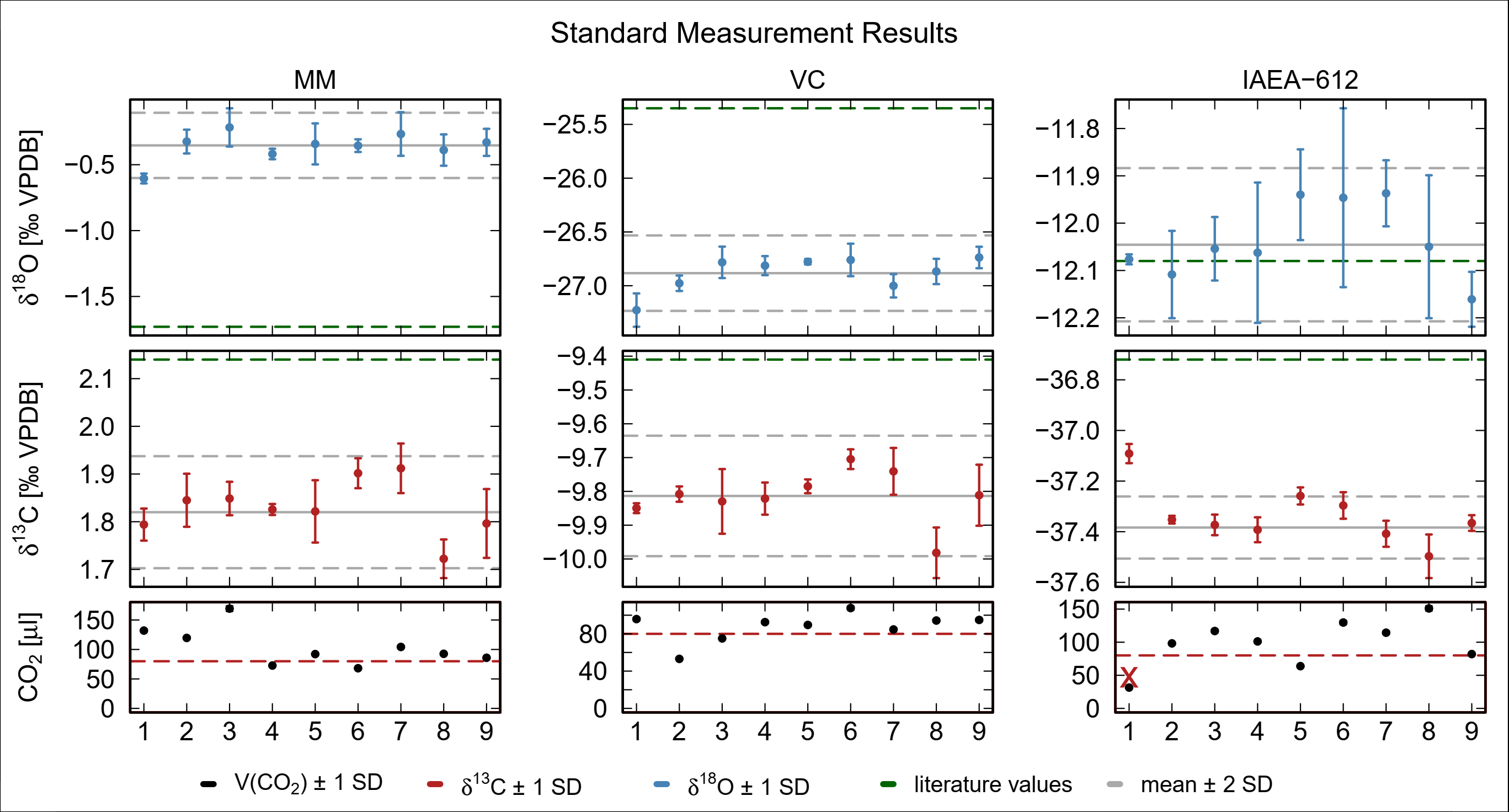


**Figure S3:** Results of the second measurement run, without without internal reference gas standards. a) δ^18^O and δ^13^C values of the RothCarbonate samples; b) δ^18^O and δ^13^C values of the carbonate standards samples. The red x’s indicate outliers which are defined in the main text


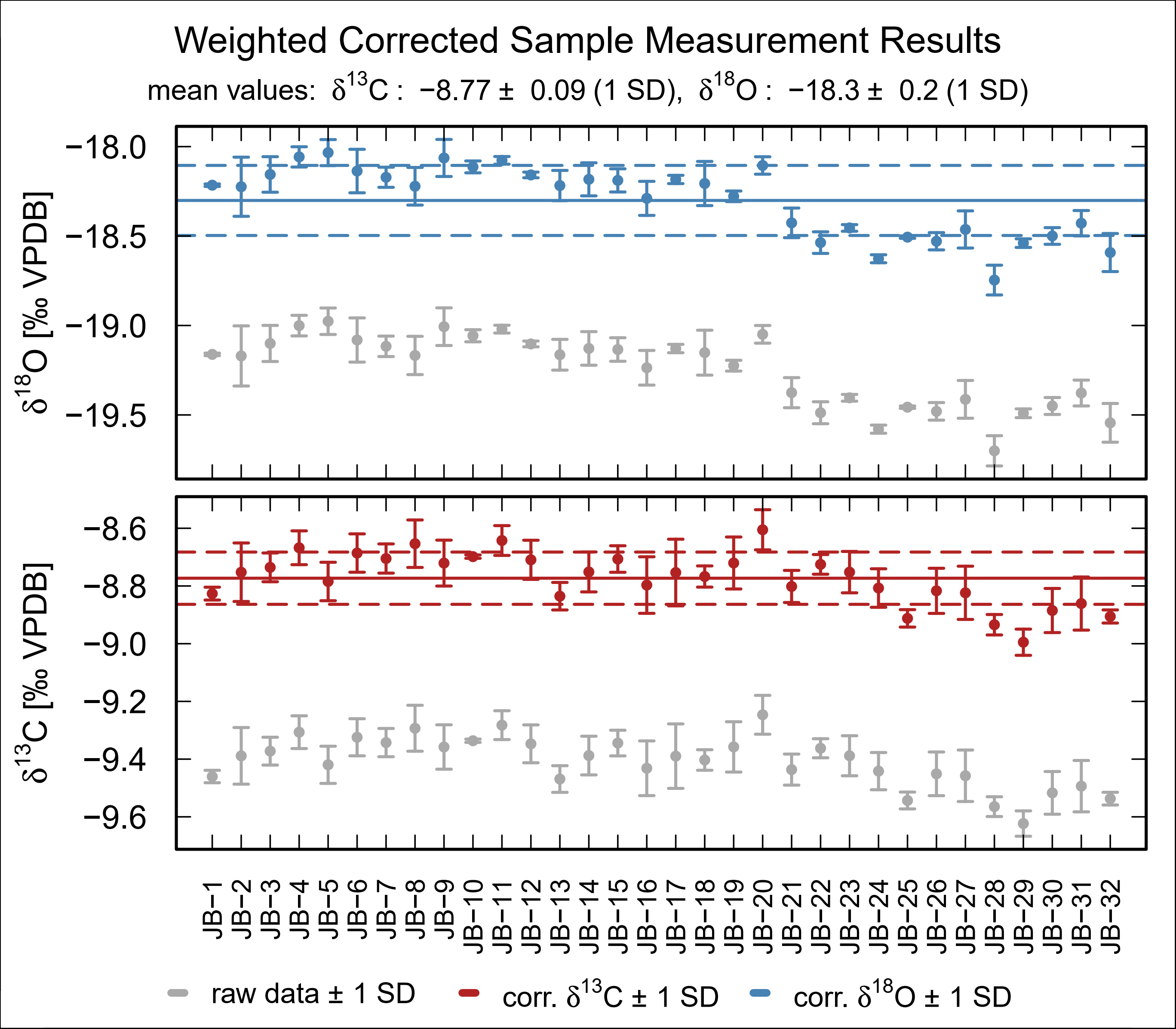

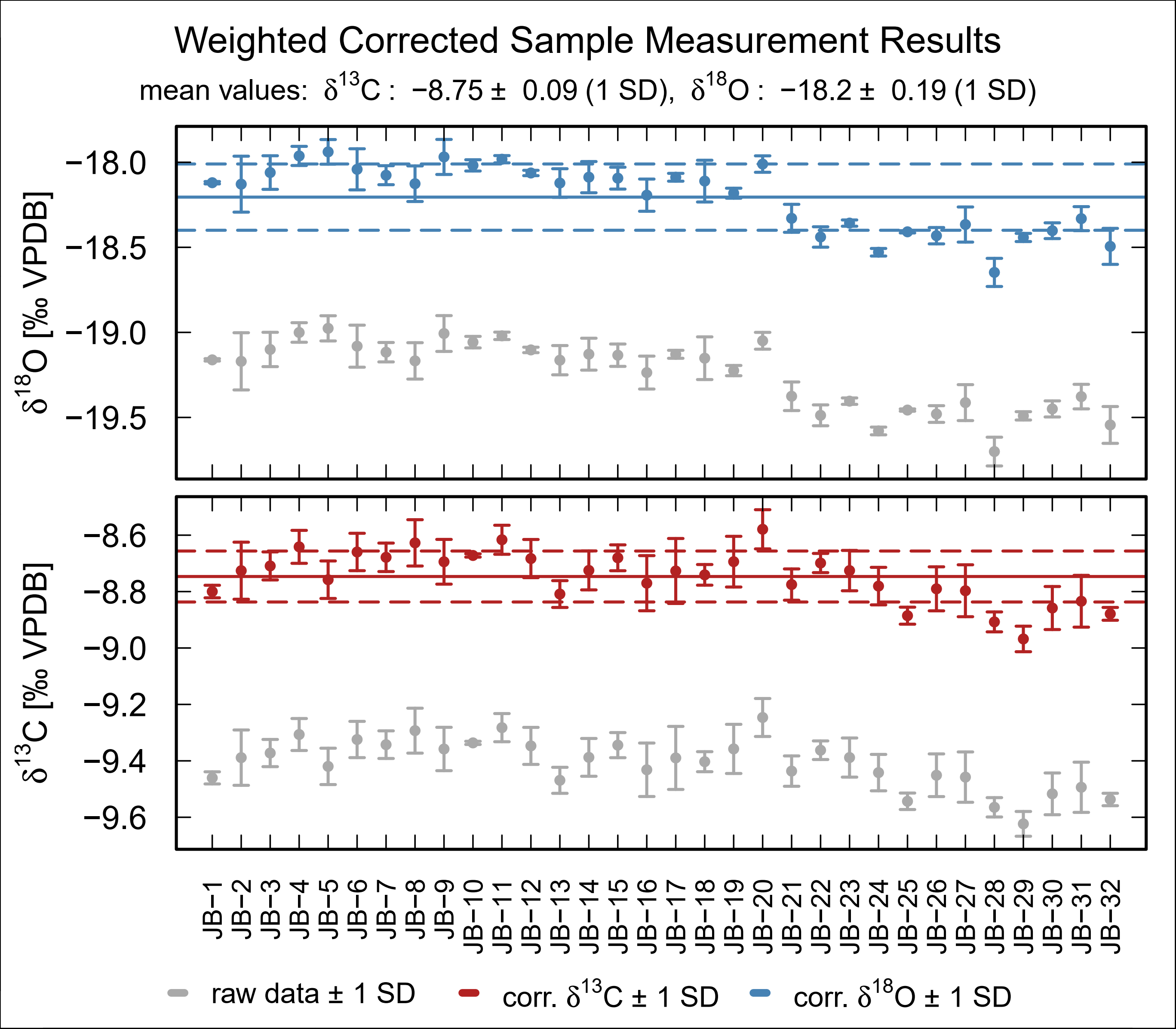

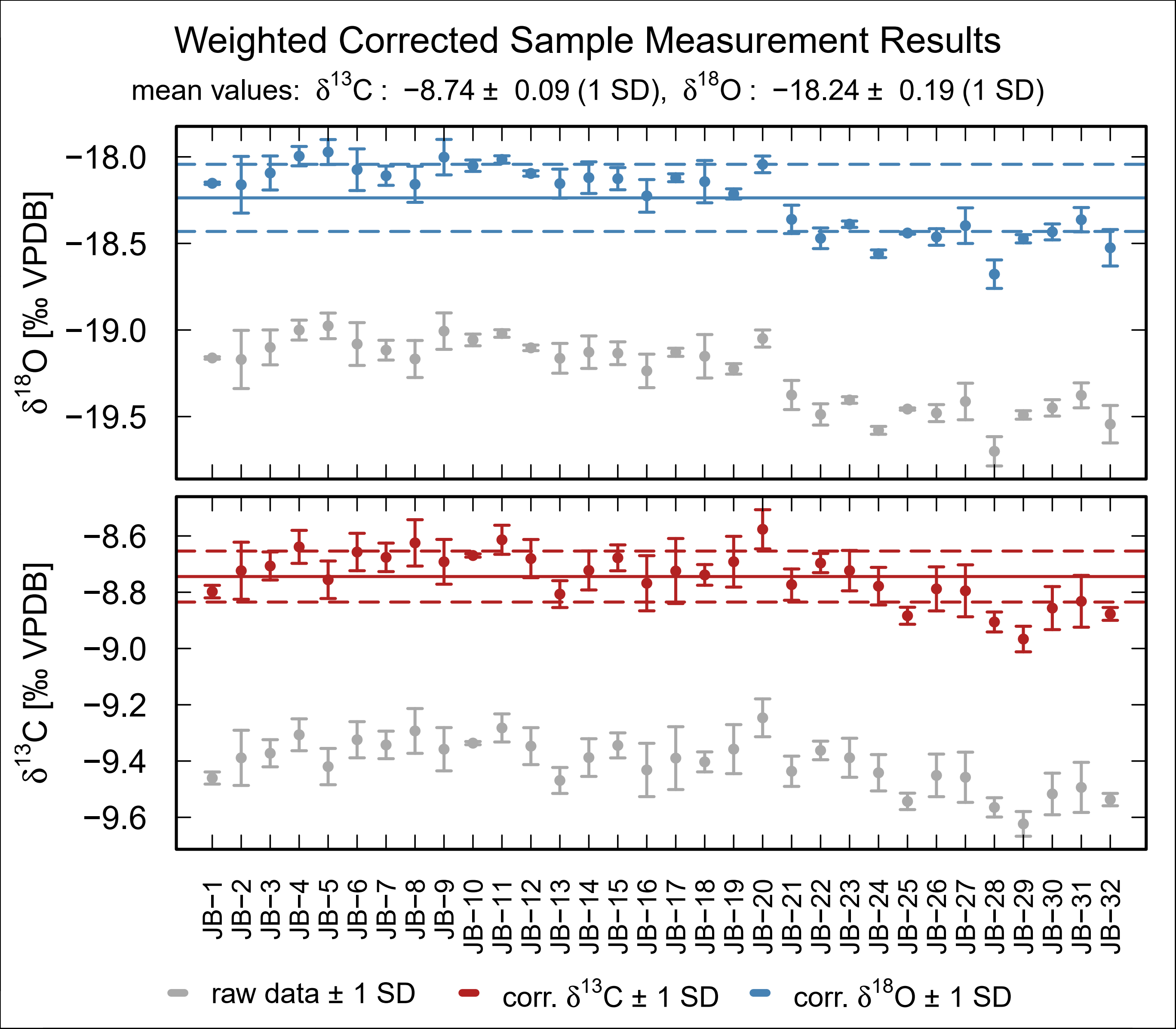


cn)

a)

bn)

**Figure S4:** Corrected δ^13^C (red) and δ^18^O (blue) values of the RC samples of the first measurement run without internal reference gas standards. Grey symbols indicate the uncorrected data. The results were calibrated applying a two-point calibration with an overall mean fit through all standards using the a) MM and VC b) MM and IAEA-612 and c) VC and IAEA-612 carbonate standards.


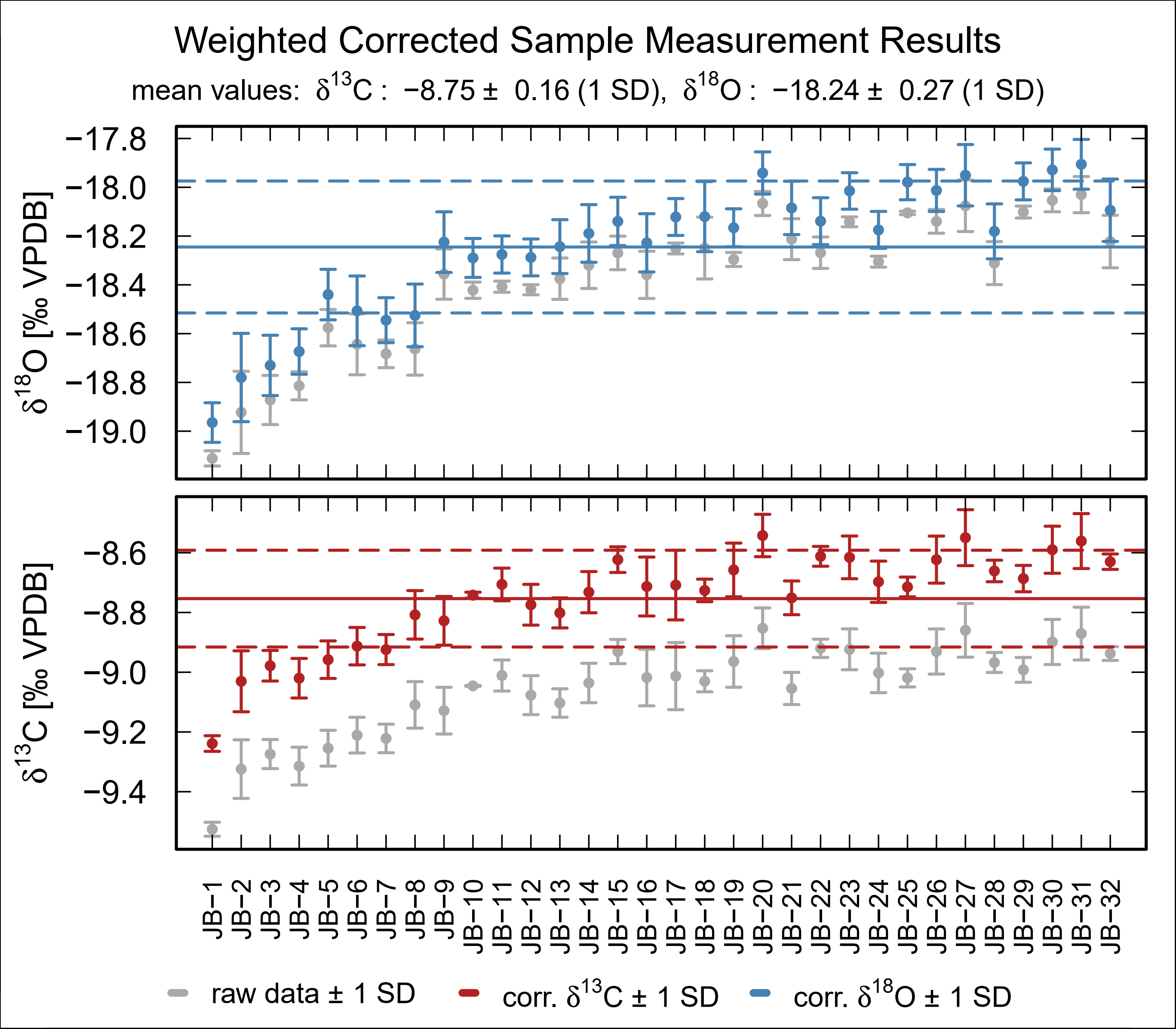

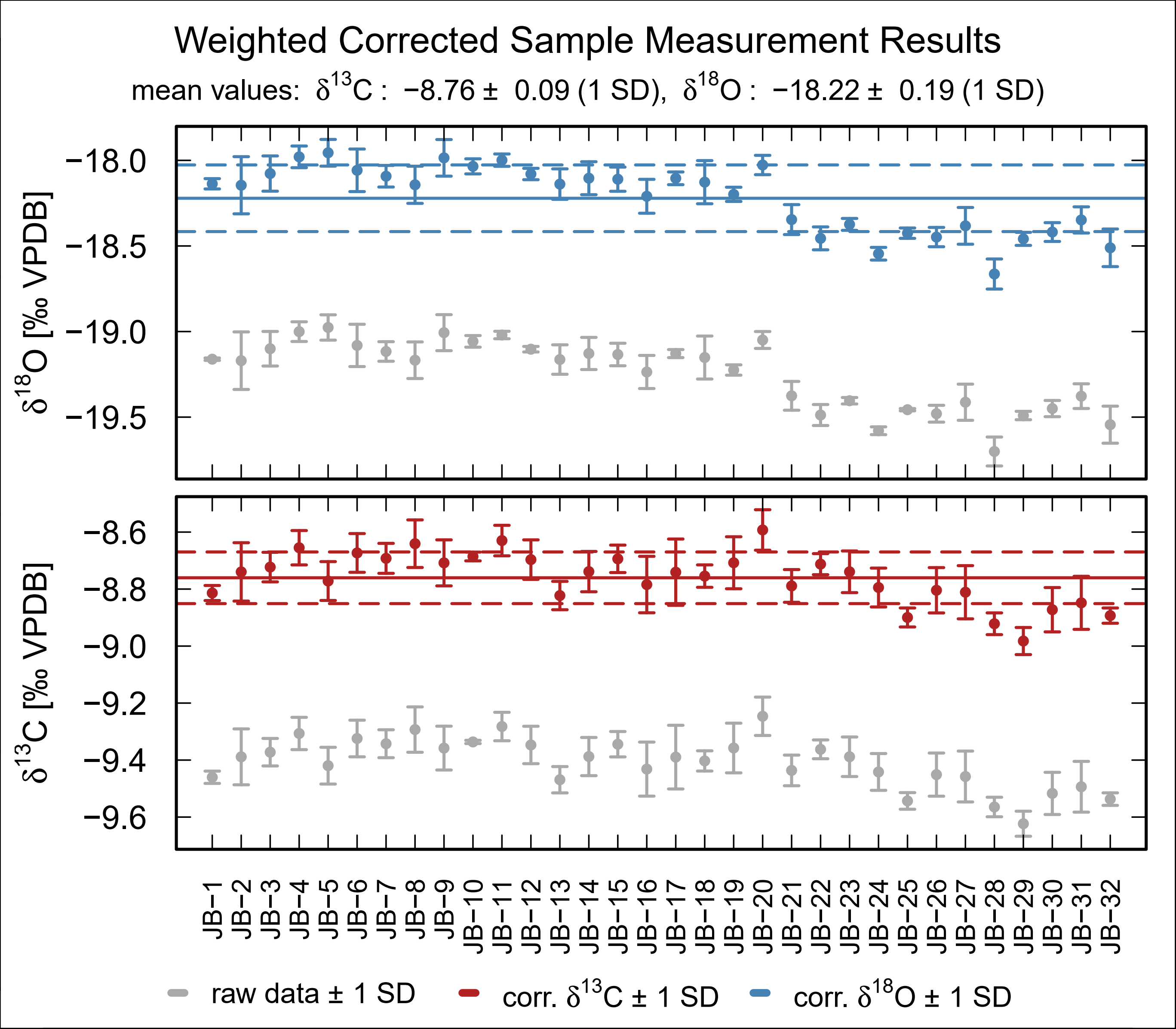


b)

a)

**Figure S5:** Corrected δ^13^C (red) and δ^18^O (blue) values of the RC samples of the first measurement run (a) with and (b) without internal reference gas standards. Grey symbols indicate the uncorrected data. The results were calibrated applying a three-point calibration with an overall mean fit through all measured standards using the MM and VC and IAEA-612 carbonate standards.


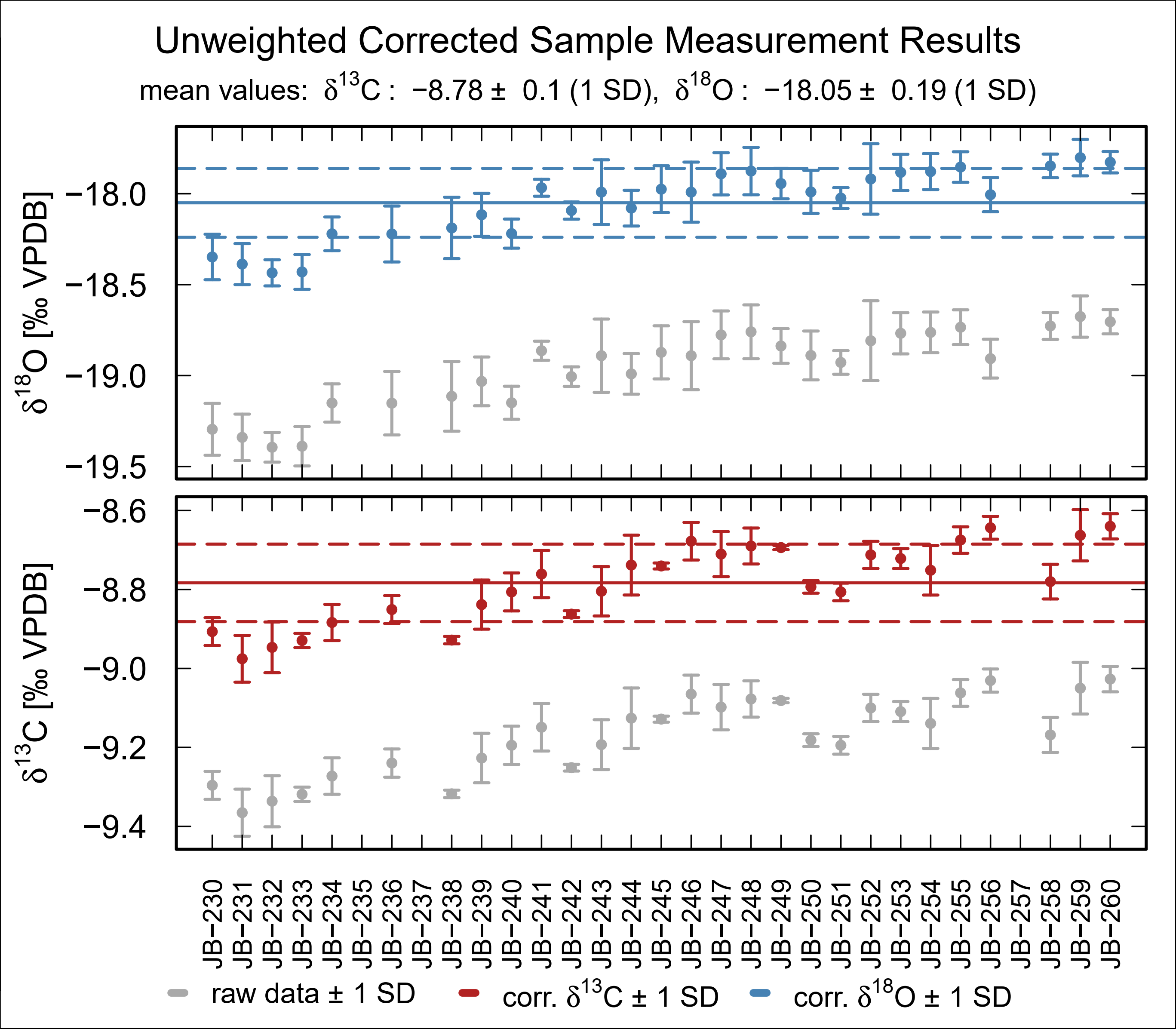

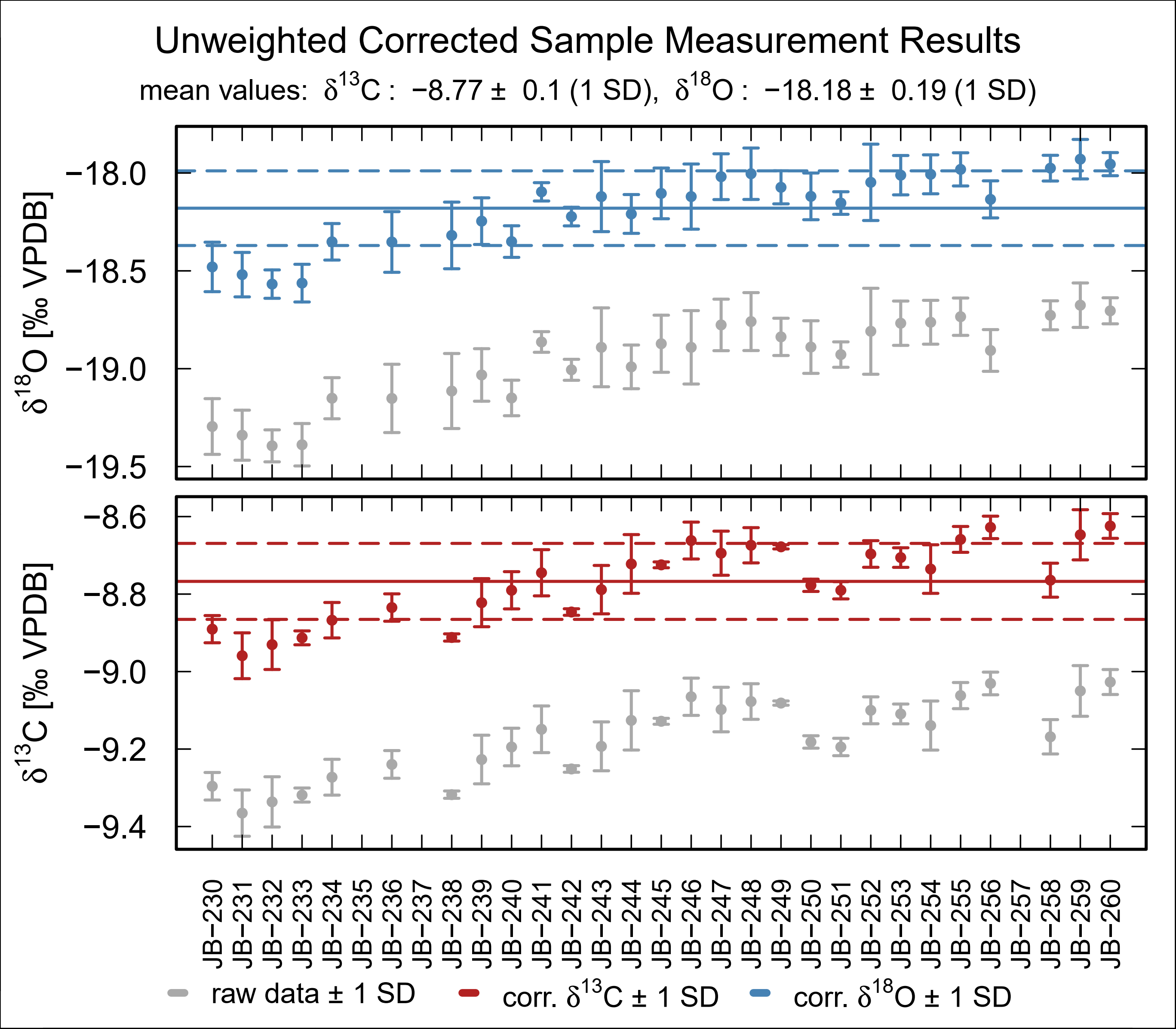

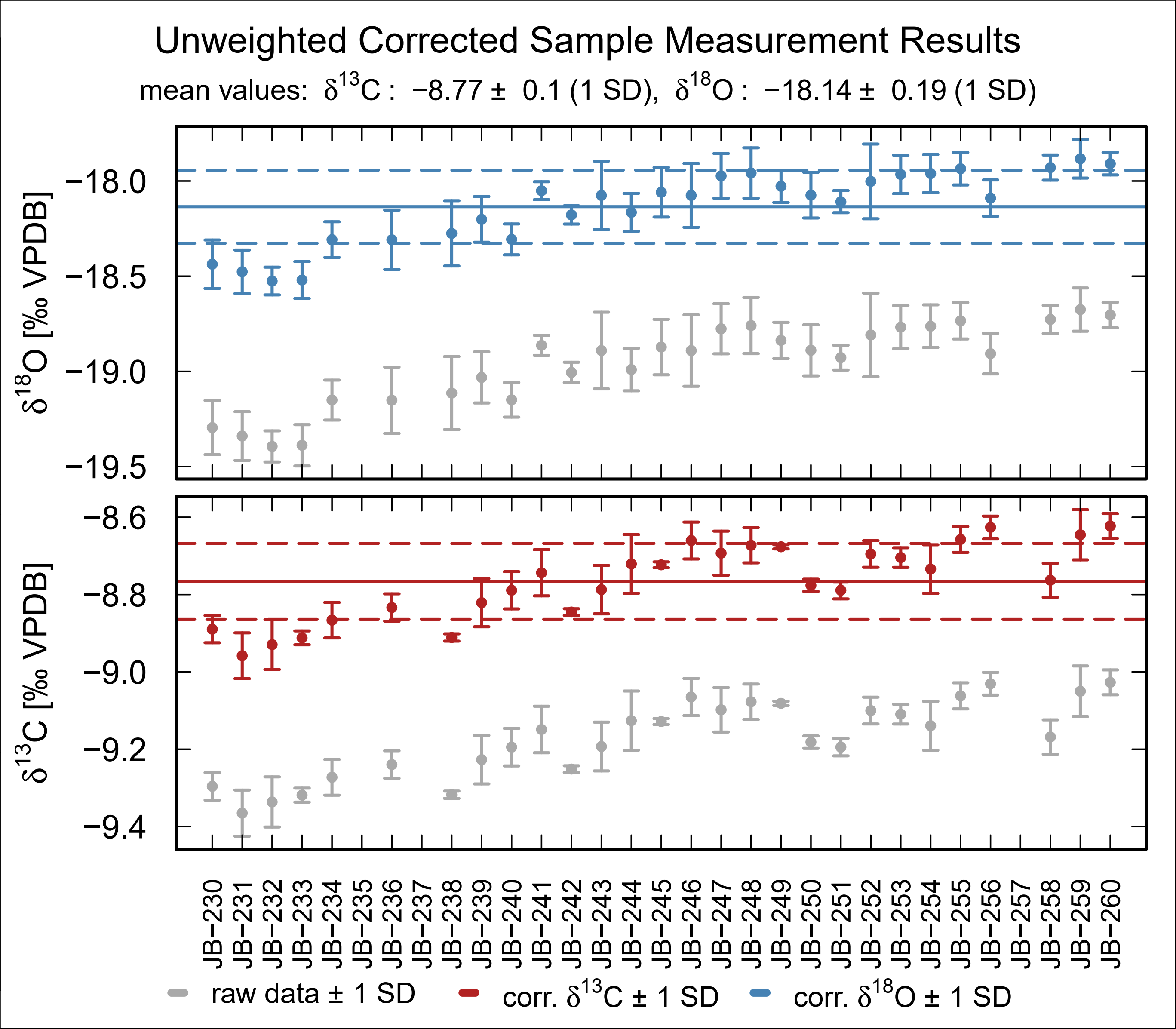


c

b)

a)

**
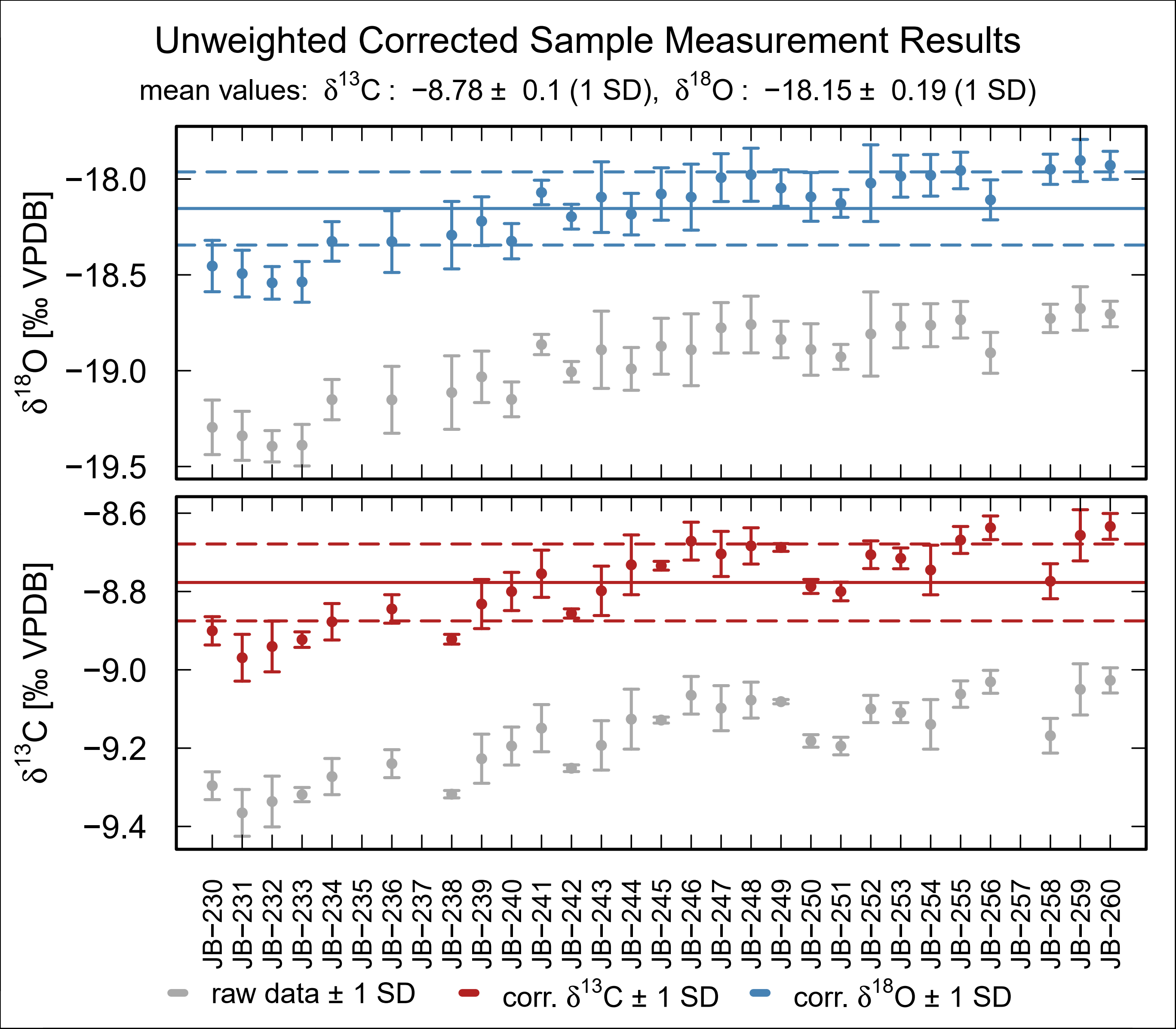
**

d

**Figure S6** Corrected δ^13^C (red) and δ^18^O (blue) values of the RC samples of the second measurement run. Grey symbols indicate the uncorrected data. The results were calibrated applying a two-point calibration with an overall mean fit through a) MM and IAEA-612, b) MM and VC c) VC and IAEA-612 and d) an overall mean fit through all three carbonate standards.


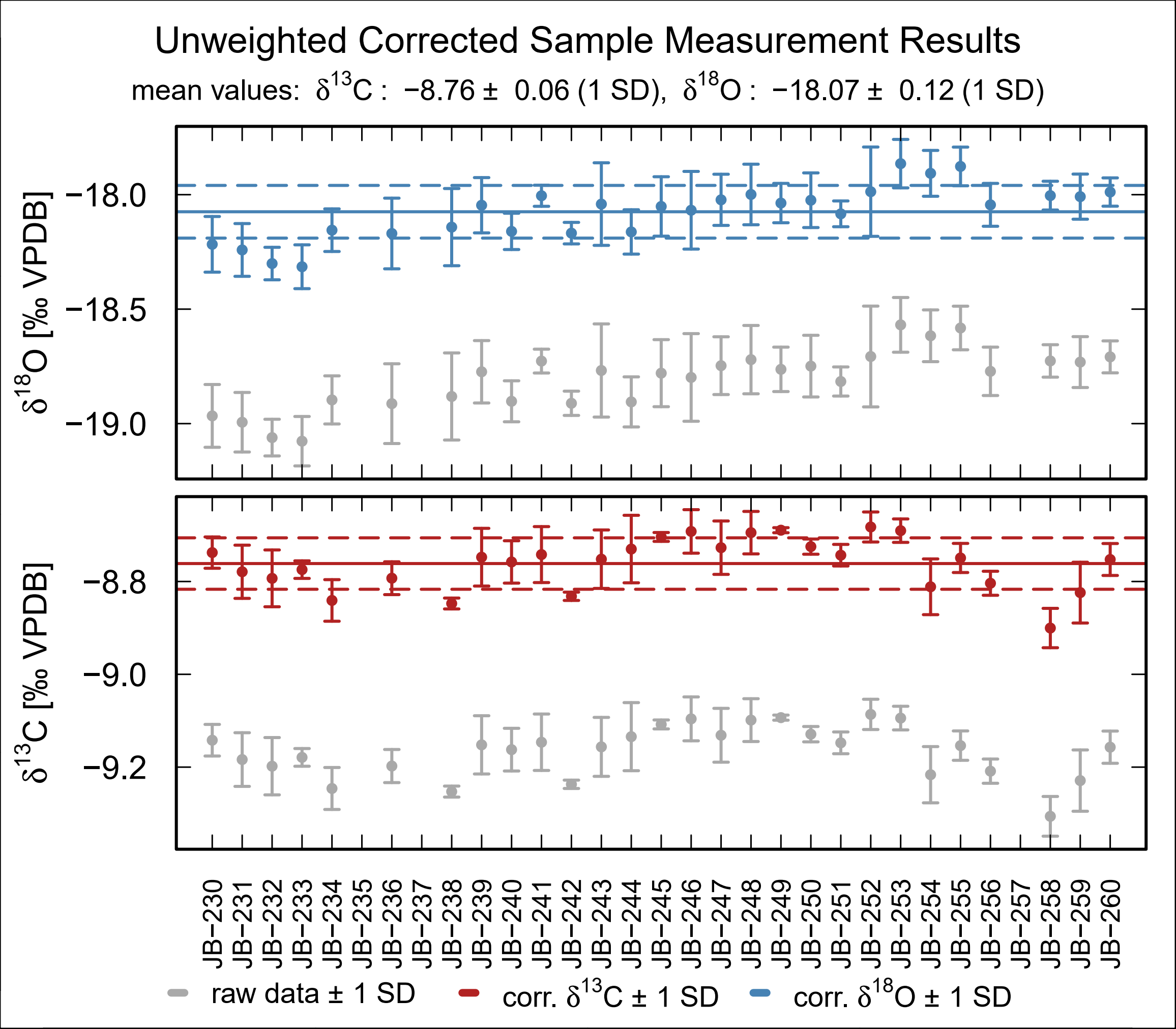

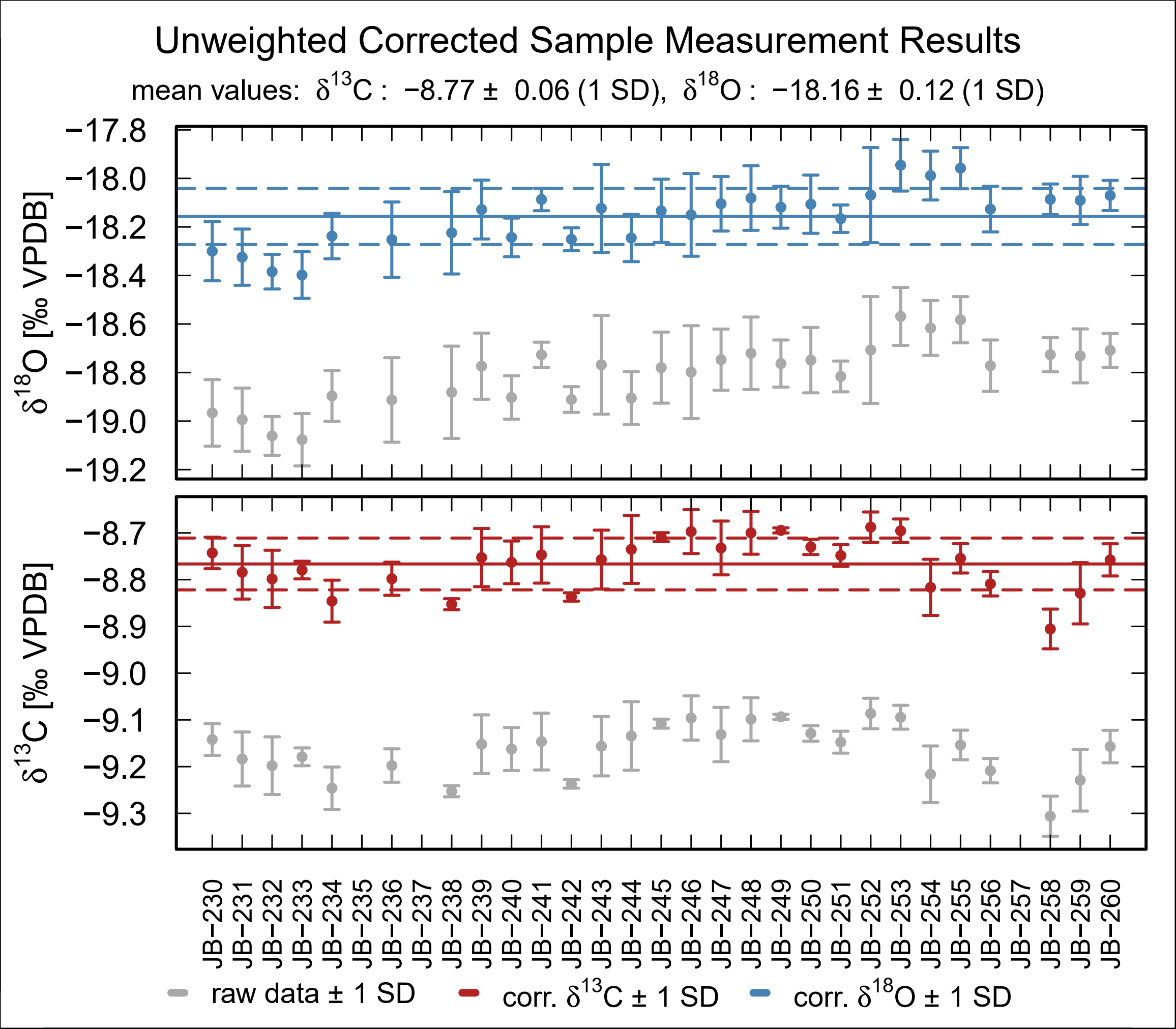

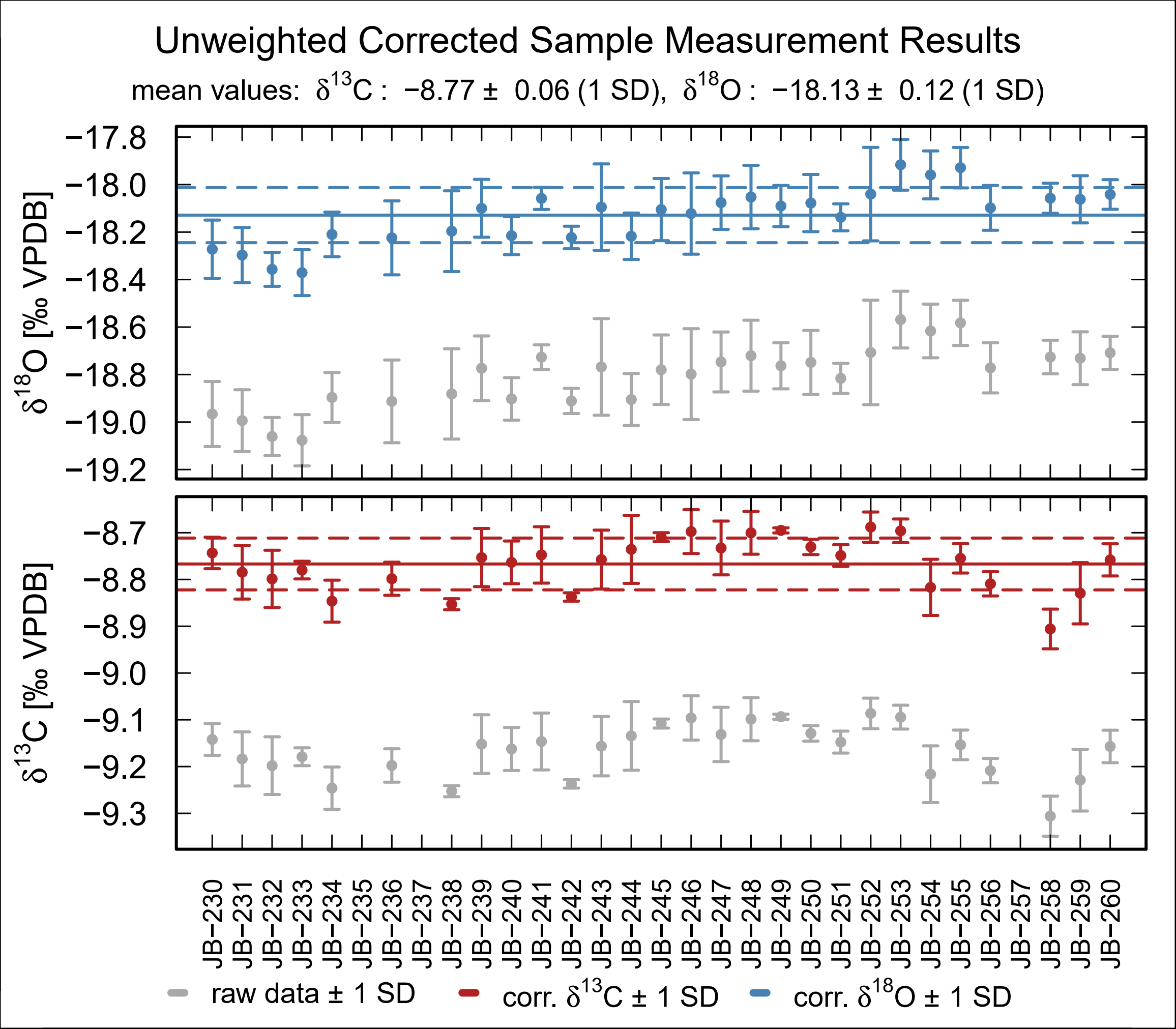


c

b

a


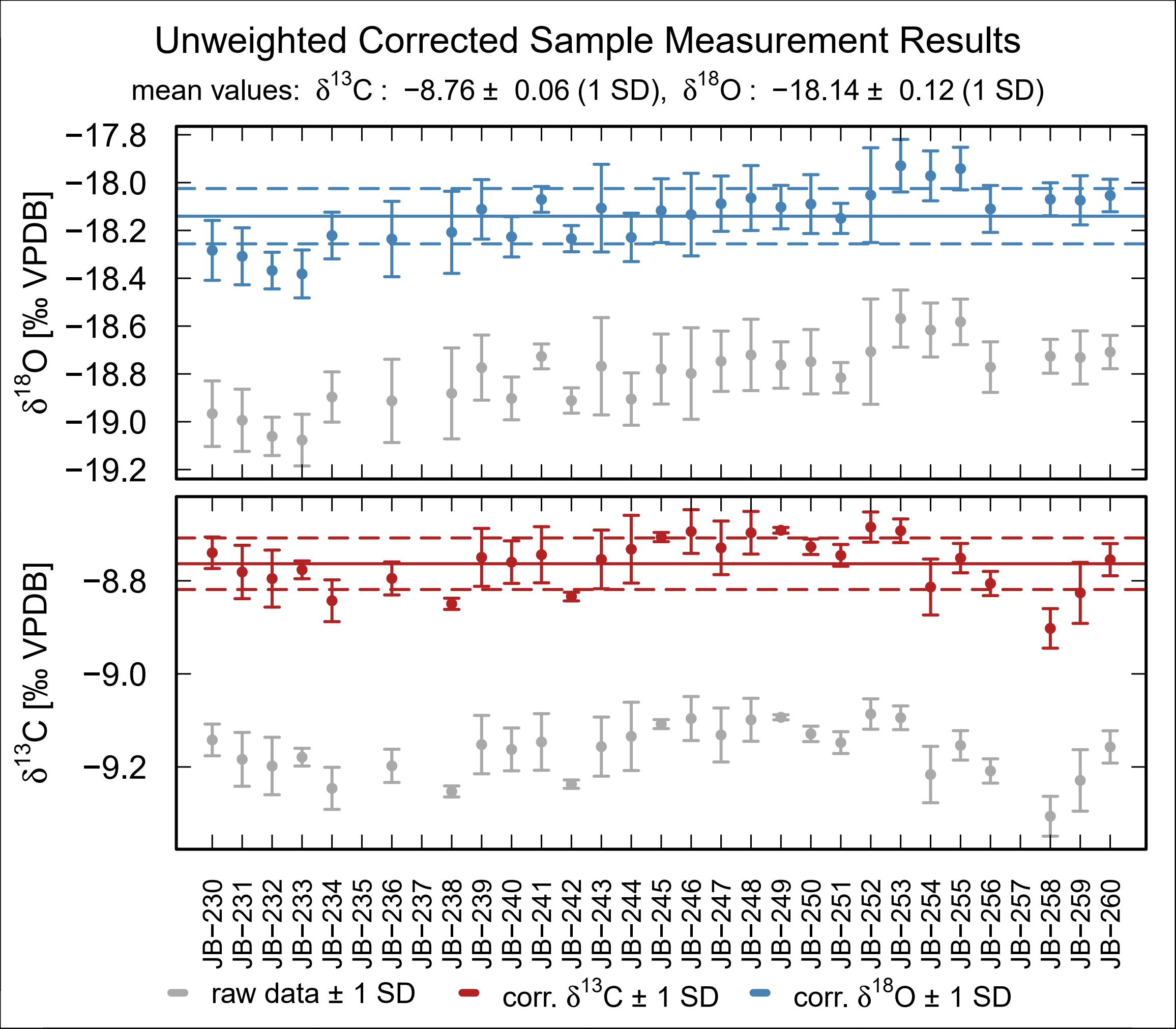


d

**Figure S7** Corrected δ^13^C (red) and δ^18^O (blue) values of the RC samples of the second measurement run without internal reference gas standards. Grey symbols indicate the uncorrected data. The results were calibrated applying a two-point calibration with an overall mean fit through a) MM and IAEA-612, b) MM and VC, c) VC and IAEA-612 and d) an overall mean fit through all three carbonate standards.


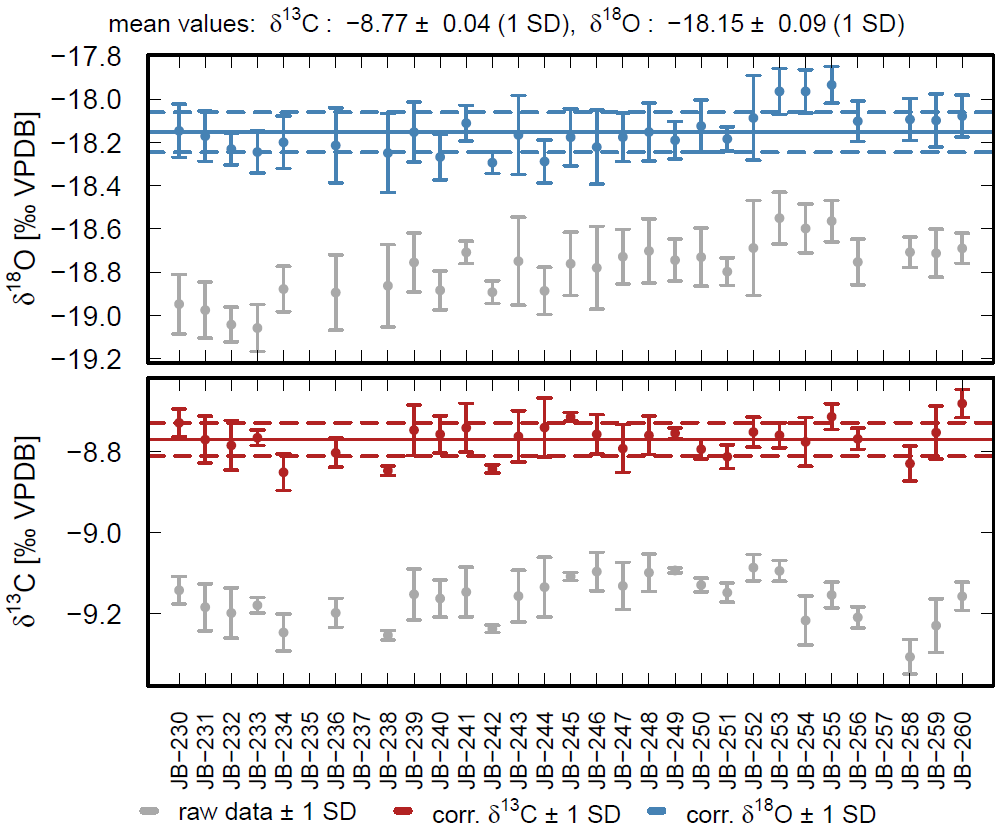


**Figure S8** Corrected δ^13^C (red) and δ^18^O (blue) values of the RC samples of the first measurement run without internal reference gas standards. Grey symbols indicate the uncorrected data. The values are calibrated against MM, VC and IAEA-612 by applying a standard bracketing.


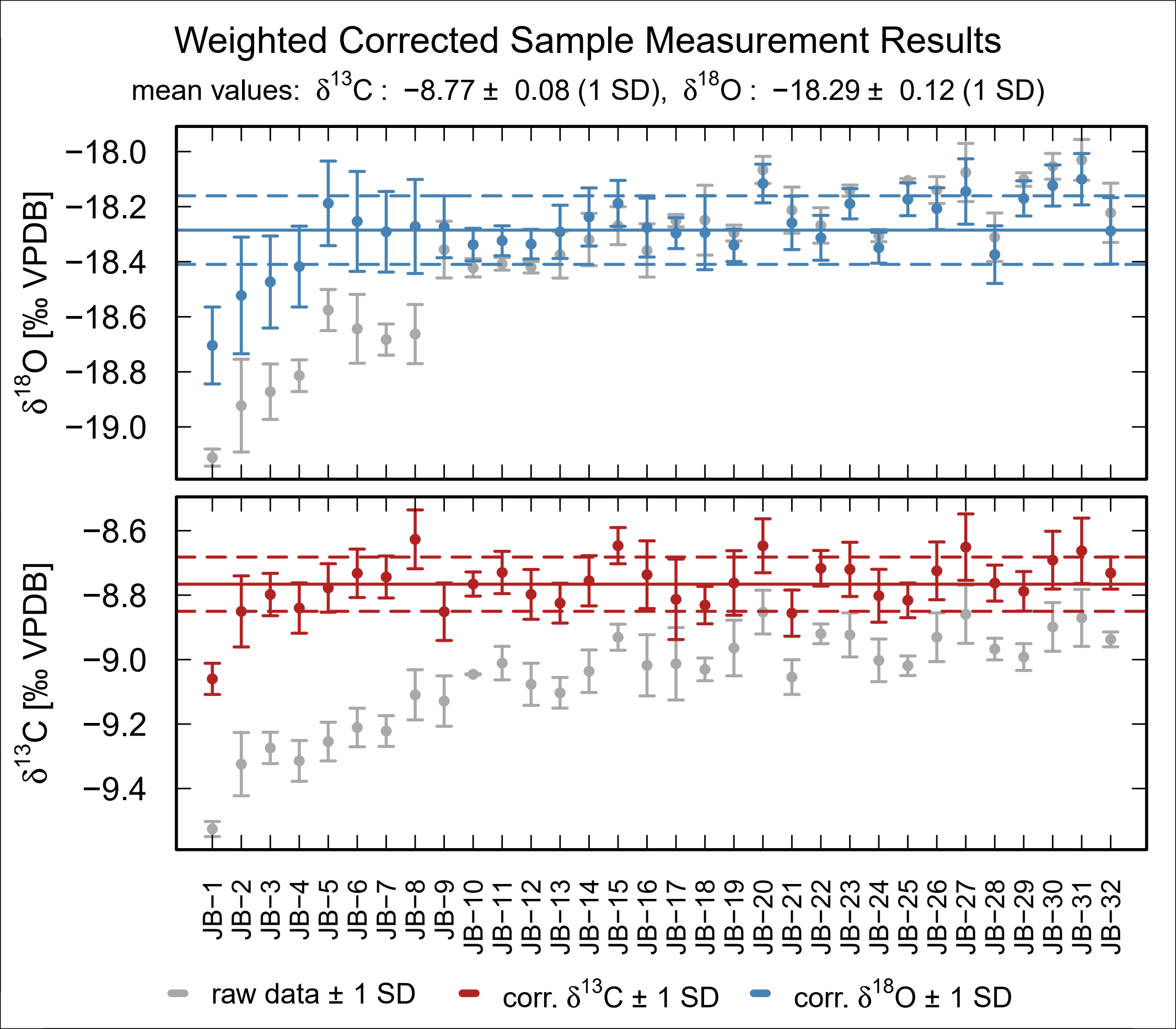


b

a


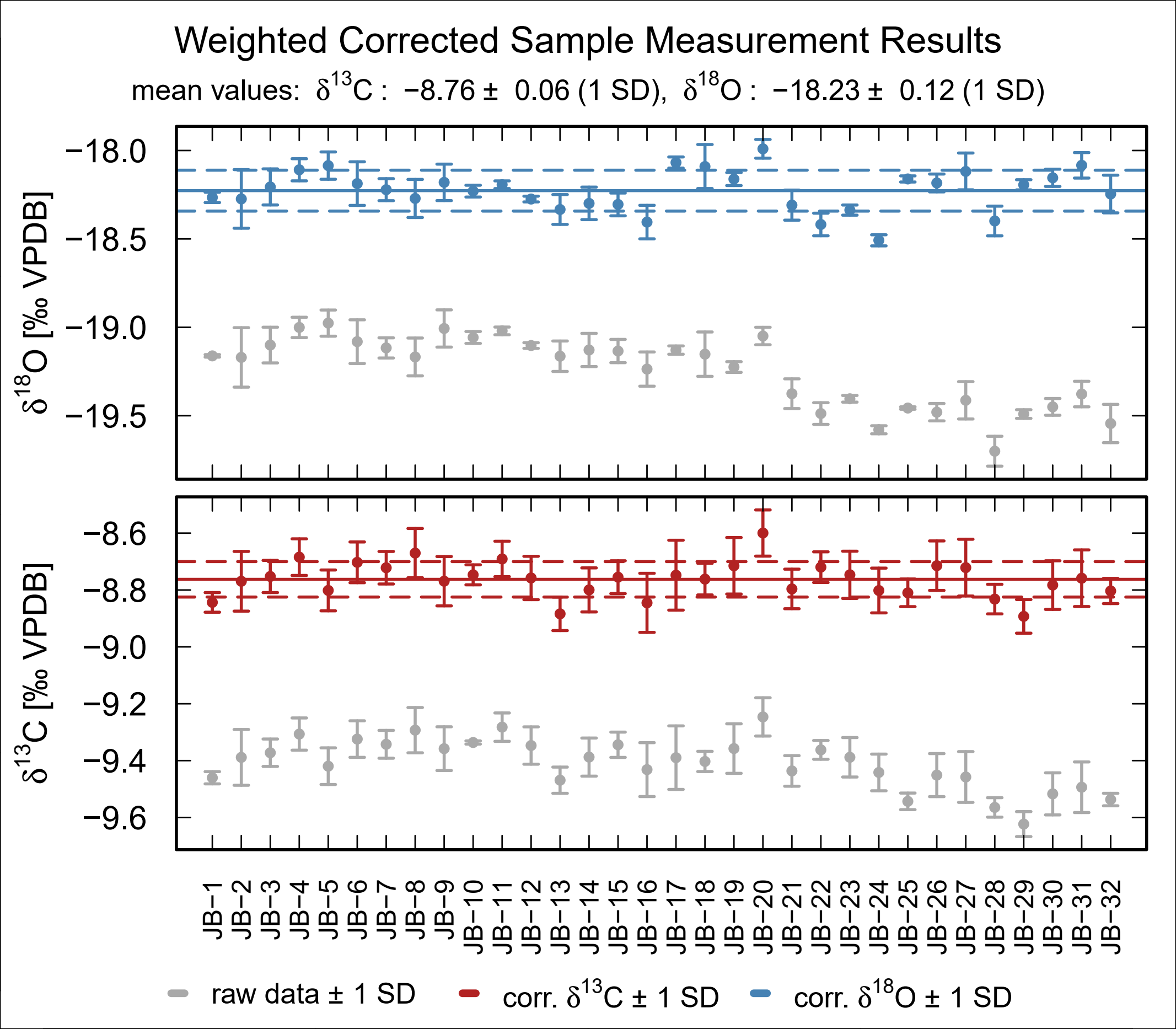


**Figure S9** Corrected δ^13^C (red symbols) and δ^18^O (blue symbols) values of the RC samples of the first measurement run but using a wider bracket of 8 samples between two sets of standards: (a) with internal reference gas standards (b) without gas standards. Grey symbols indicate the uncorrected data. The values are calibrated against MM, VC and IAEA-612 by applying a standard bracketing.


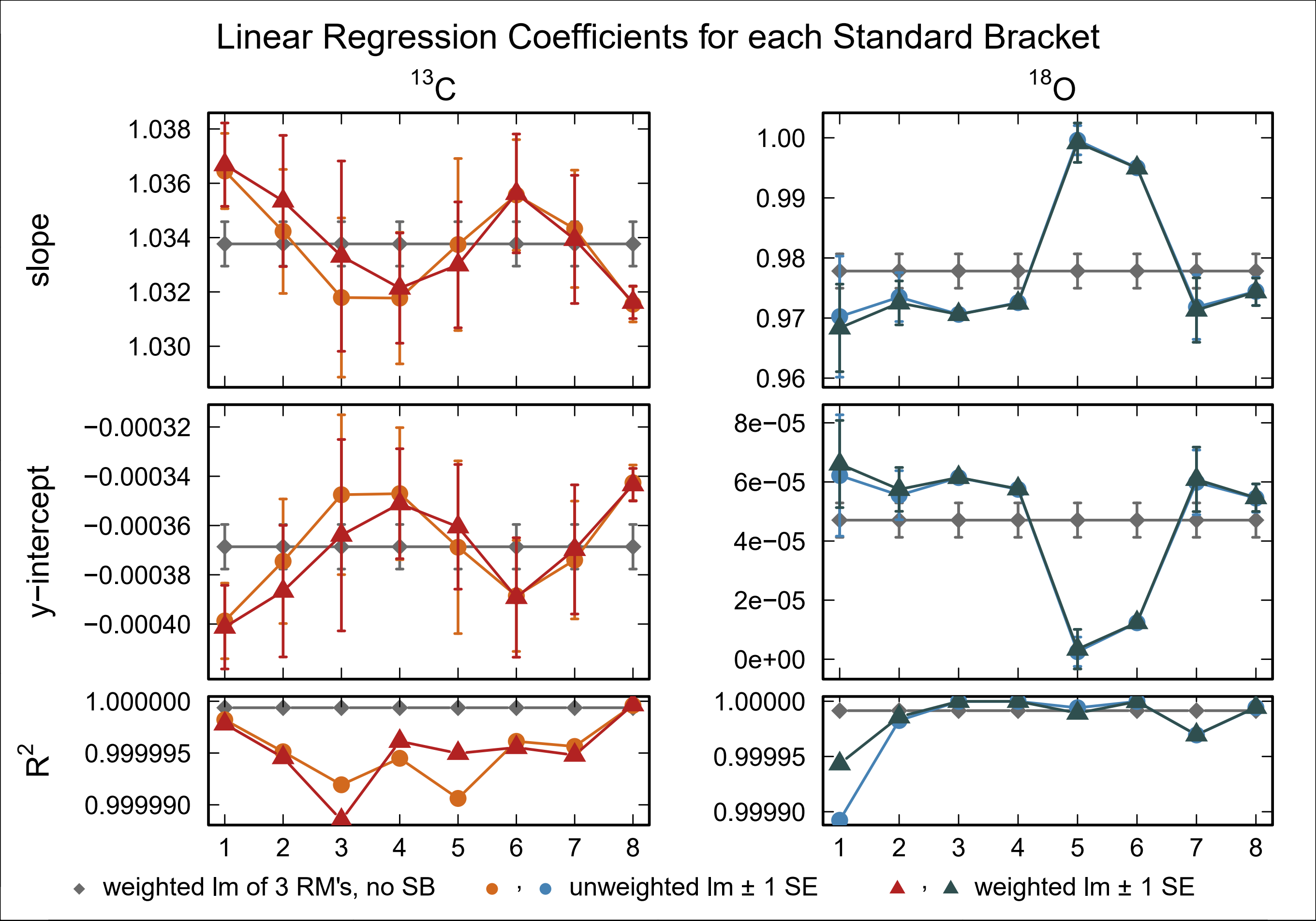


**Figure S10** Linear regression coefficients for the first measurement run, without reference gas standards. The colored symbols indicate the individual correction coefficients for every individual standard block obtained from the standard bracketing (triangles weighted lm, circles unweighted lm), the grey diamonds indicate the coefficients of the overall mean fit of the three reference materials.

a


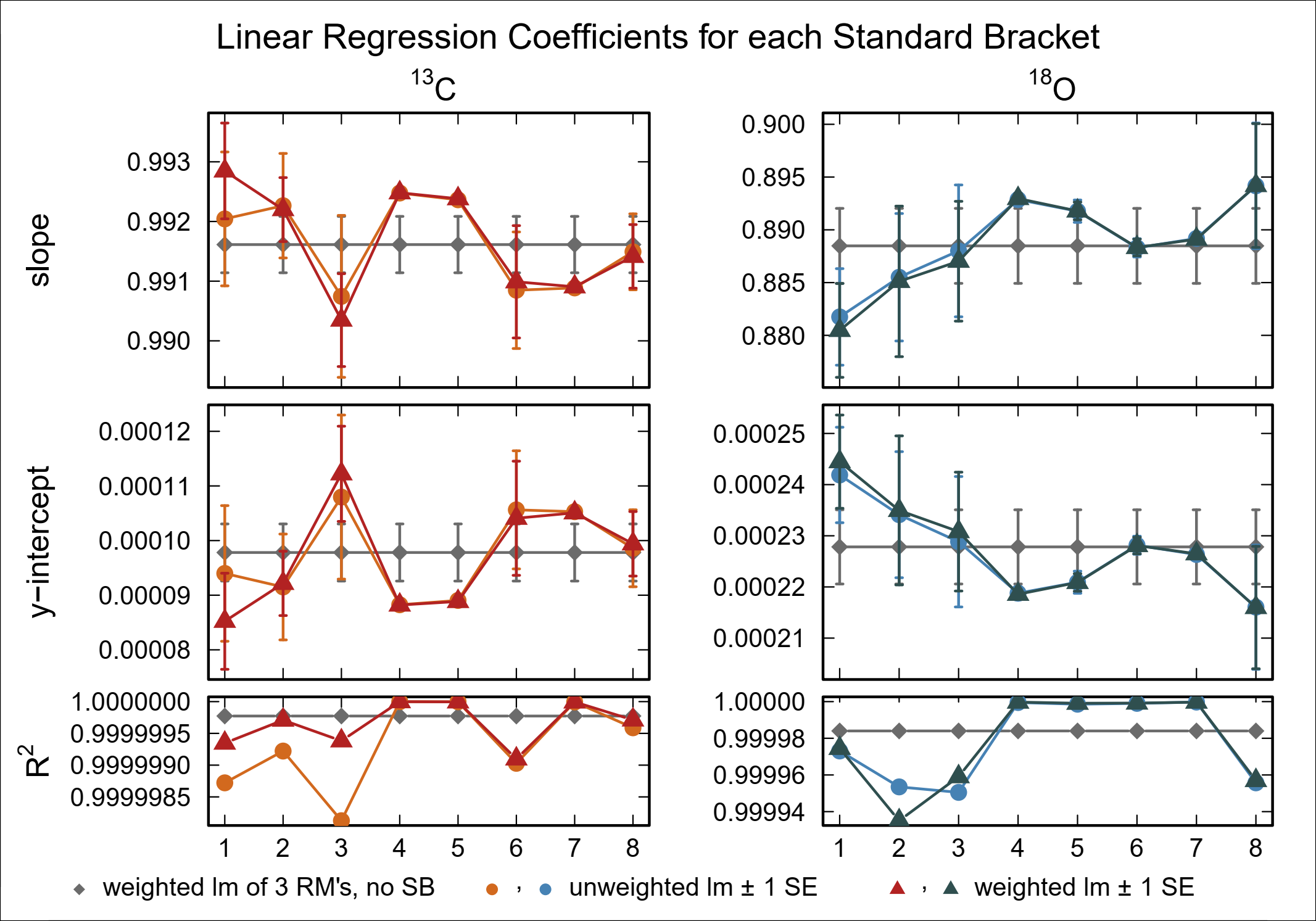


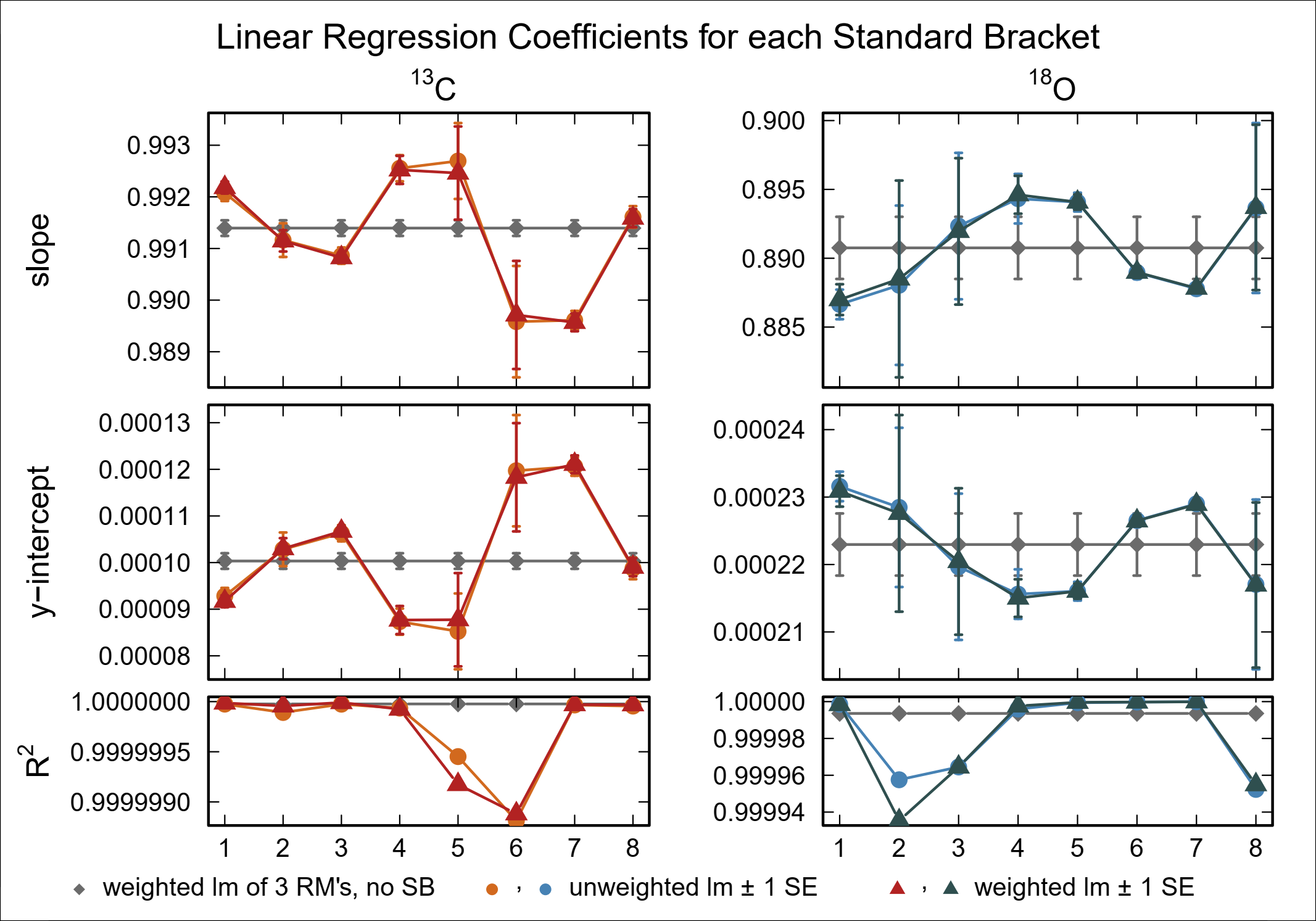


b

**Figure S11** Linear regression coefficients for the second measurement run, (a) internaly calibrated against the reference gas standards (b) without internal reference gas standards. The colored symbols show the regression coefficients for the individual standard blocks obtained from the standard bracketing technique (triangles show the weighted regression, circles the unweighted regression). The grey diamonds show the average coefficient obtained by the three-point calibration.

a


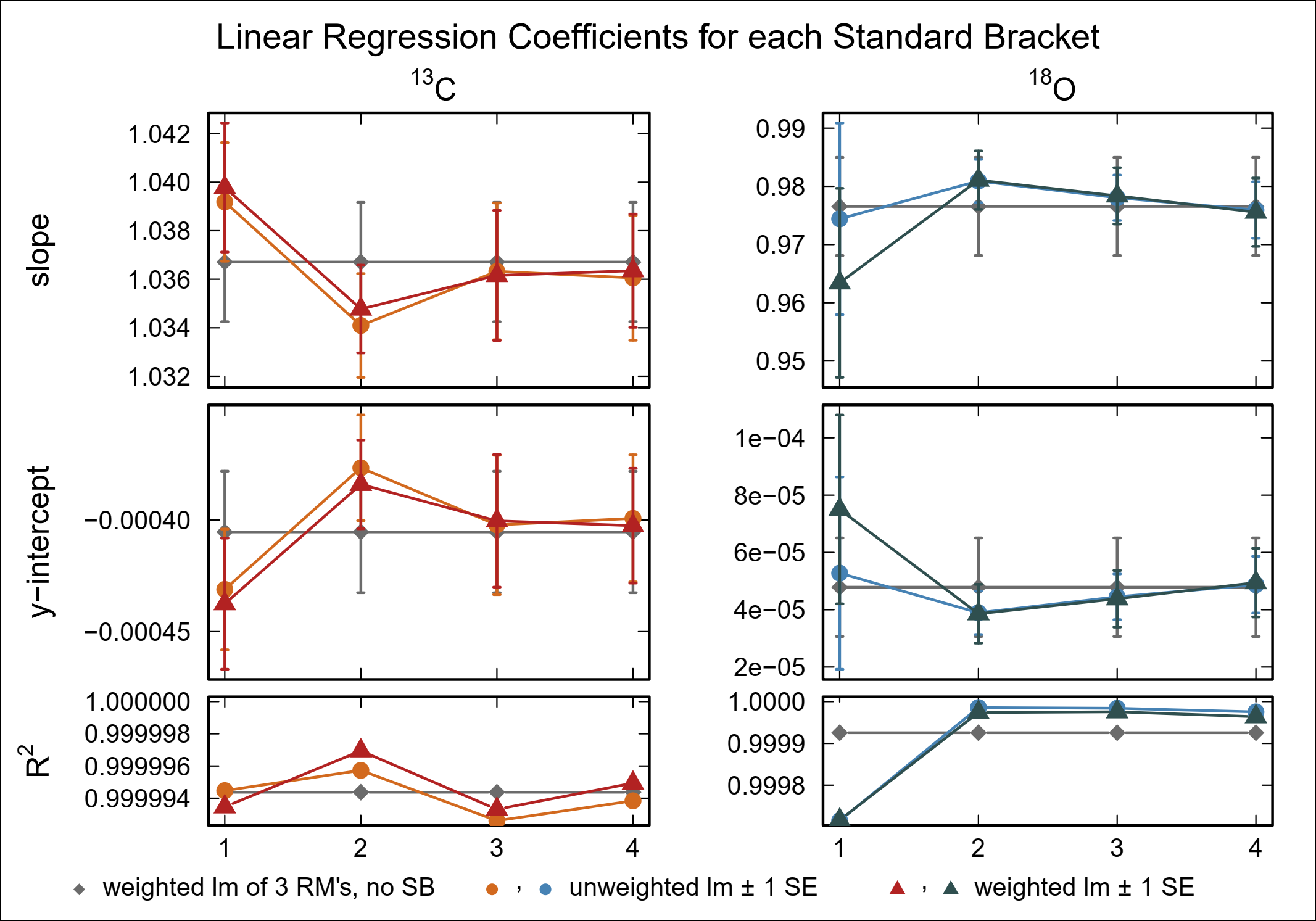


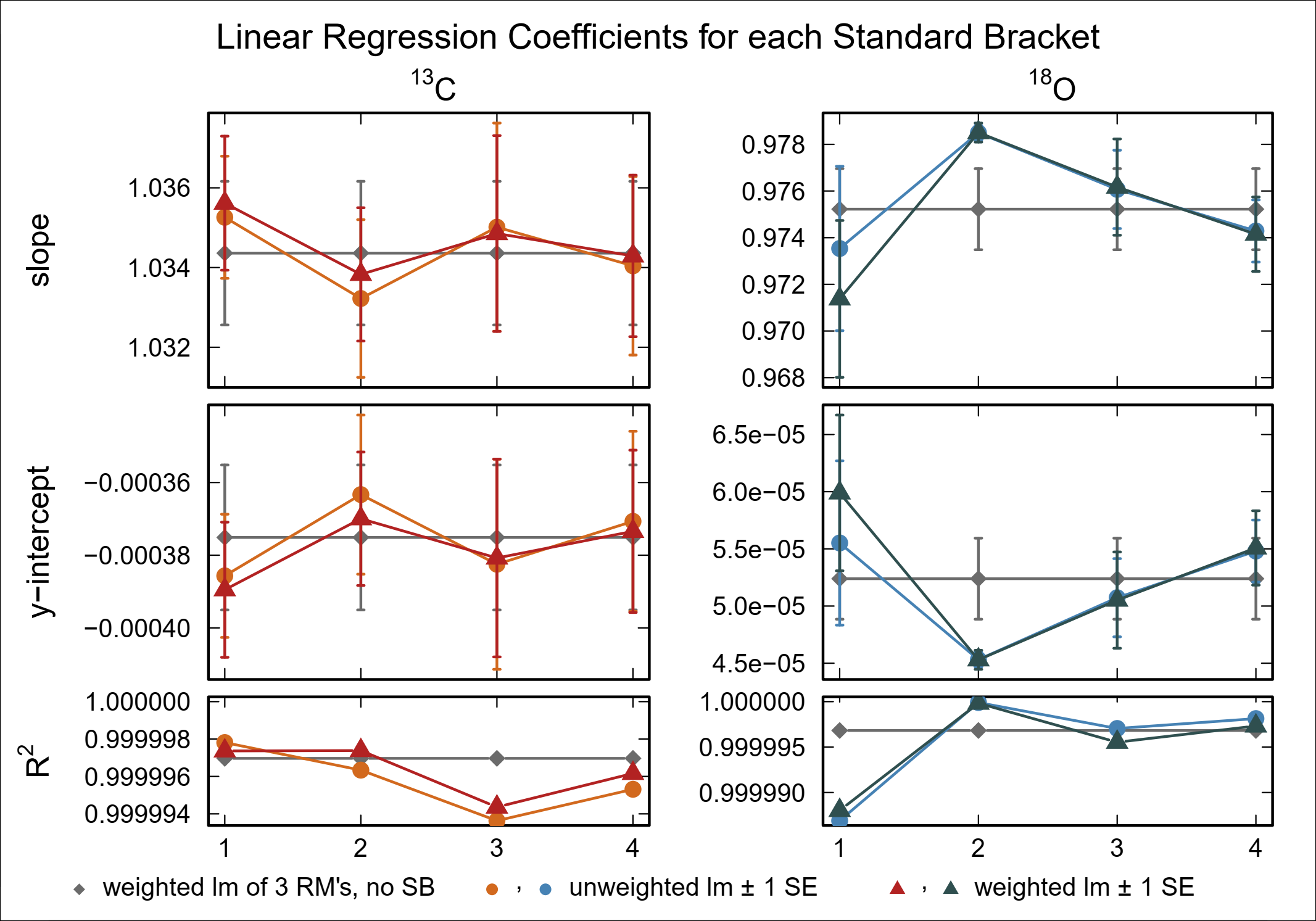


b

**Figure S12** Linear regression coefficients for the first measurement run but with a wider bracket width of 8 samples between two blocks of carbonate standards, (a) internaly calibrated against the reference gas standards (b) without internal reference gas standards. The colored symbols show the regression coefficients for the individual standard blocks obtained from the standard bracketing technique (triangles show the weighted regression, circles the unweighted regression). The grey diamonds show the average coefficient obtained by the three-point calibration.
