# Supplementary material for: Stable Carbon and Oxygen Isotope Analysis of Carbonates and DIC Using the Delta Ray Isotope Ratio Infrared Spectrometer (IRIS): Precise and Accurate Measurements Applying a 3‐Point Calibration and Standard Bracketing
Source: Rapid Commun Mass Spectrom. 2025 Dec 29;40(6):e70021. doi: 10.1002/rcm.70021 (PMC12748363; doi:10.1002/rcm.70021)
Supplement: Supplementary file 2 — Data S1: Supporting Information. [file RCM-40-e70021-s004.docx]

**Manual for the R code provided to the Manuscript “Stable carbon and oxygen isotope analysis of carbonates and DIC using the DeltaRayTM Isotope Ratio Infrared Spectrometer (IRIS): precise and accurate measurements applying a three-point calibration and standard bracketing”**

**How to properly design the measurement run in order to be able to evaluate it with the provided R Skript**

- All measurements of the same carbonate standard within one experiment have to be named the same in Qtegra.
- E.g. “IAEA-612” for all measurements of the carbonate reference material IAEA-612
- Carbonate samples should be named according to the following scheme:
  - “Sample name” + delimiter + ascending ID
  - E.g. “MH-1”, “MH-2”, “MH-3”, etc. or “JB-230”, “JB-231”, “JB-232”, etc.
- The measurement of a “dummy” sample before the first measurement of a carbonate standard is recommended. This “dummy” sample should be named differently than the other samples.
- The number of sample measurements between the measurement of carbonate standards may vary but needs to be specified in the R script. In order for the R script to properly calibrate the sample measurements, the three used carbonate standards need to be measured directly successively.
- An exemplary measurement run with correct naming might look as follows:
  - Experiment name: “Experiment_carbonates_test.csv”
  - “Dummy”, “MM”, “VC”, “IAEA-612”, “Sample-1”, “Sample-2”, “Sample-3”, “Sample-4”, “MM”, “VC”, “IAEA-612”, “Sample-5”, “Sample-6”, “Sample-7”, “MM”, “VC”, “IAEA-612”
  - This experiment would consist of one “dummy” and seven measured samples, which are divided into two sample brackets and bracketed by the carbonate standards MM, VC, IAEA-612.

**Preparation**

- Please export the resulting .xlsx file, generated by QTegra after the measurement sequence is finished, as an UTF-8.csv document, so that the R script can handle it.
- The latest version of R ^1^ and the packages Hmisc^2^, tcltk^1^, openxlsx^3^ and extrafont^4^ need to be installed. The use of RStudio^5^ is recommended.
- The R scripts “DeltaRay_calibration_software_user.R” as well as “DeltaRay_calibration_functions_user.R need to be in the same directory as the .csv-file of the experiment
- To evaluate the data, “DeltaRay_calibration_software_user.R” needs to be opened in a preferred R editor.

**The following lines of code need to be adjusted by the user:**

- **Lines 19, 20**
  - These two commands need to be executed once before the first use of the code to properly generate the .pdf-files.
- **Lines 26, 28:**
  - Specify the directories from which a) the .csv-file should be loaded and b) in which the calibration results should be saved.
- **Lines 36 to 65:**
  - Enter the parameters which describe the performed experiment. For the example mentioned above the code should look like follows:

**
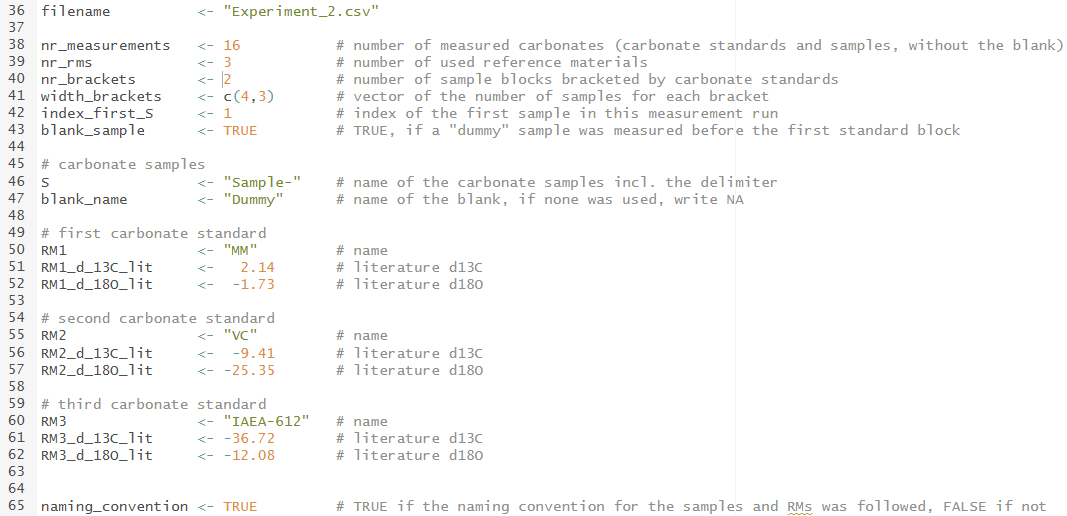
**

- **Lines 73 to 81:**
  - Define the literature constants, as well as the temperature, at which the carbonate reaction took place.

**From line 82 on the whole script can be executed**

- The process of the data calibration is performed automatically, based on the preset parameters.
- Two R Graphics windows will open:
  - The original sample measurement results
  - The original carbonate standard measurement results
- A question window will open, asking whether the removal of any sample measurement is desired
  - If YES, another graphics window displaying the sample measurements will pop up. Any sample can be removed from the dataset by clicking on its respective CO_2_-content results (red-bordered area of the plot). A red X will appear next to the selected values. After all selected samples have been indicated, the evaluation will continue after clicking on “ending” → “ending locator()”.
  - If N, the evaluation continues with no further changes.
- A question window will open, asking whether the removal of any carbonate standard measurement is desired.
  - If YES, another graphics window displaying the carbonate standard measurements will pop up. Any standard can be removed from the dataset by clicking on its respective CO_2_-content results (red-bordered area of the plot). A red X will appear next to the selected values. The removal-process starts with the first carbonate standard. To select measurements of the second and the third carbonate standard, “ending” → “ending locator()” needs to be clicked. After all selected samples have been indicated, the evaluation will continue after clicking on “ending” → “ending locator()”.
  - If NO, the evaluation continues with no further changes.
- The data evaluation is performed and more R Graphics windows will open:
  - Plots showing the sample measurement results without the previously removed samples as well as the carbonate standard measurement results with interpolated values for the previously removed standards.
  - A plot showing the calibrated measurement results.
  - A plot showing the linear regression coefficients for each standard bracket, which were used for data calibration.
- A .xlsx-file is generated and saved in the previously defined directory, which contains three worksheets:
  - The original carbonate standard measurement results, as well as standard values used for data calibration.
  - The original sample measurement results, as well as the calibrated measurement results.
  - The linear correction coefficients used for data calibration.
- The following plots will be saved as .pdf-files:
  - The original and corrected sample measurement results
  - The original carbonate standard measurement results as well as the carbonate standards used for data calibration
  - The linear regression coefficients used for data calibration.

**References**

- 1. RCoreTeam. R: A language and environment for statistical computing. . *R Foundation for Statistical Computing, Vienna, Austria.* 2025.
- 2. Harrel Jr. FE, Dupont, Charles. Hmisc: Harrell Miscellaneous. R-Packege version 5.2-4. 2025.
- 3. Schauberger PW, Alexander; Braglia, Luca; Sturm, Joshua; Garbuszus, Jan Marvin; Barbone, Jordan Mark, Zimmermann, David; Kainhofer, Reinhold. openxlsx: Read, Write and Edit xlsx Files. 2025.
- 4. Chang WB, Frederic. extrafont: Tools for Using Fonts. 2025.
- 5. team P. RStudio: Integrated Development Environment for R. *Posit Software, PBC, Boston, MA.* 2025.
